# Single-cell RNA-sequencing highlights a curtailed NK cell function in convalescent COVID-19 pregnant women

**DOI:** 10.3389/fimmu.2025.1560391

**Published:** 2025-06-30

**Authors:** Madhuri S. Salker, Nor Haslinda Abd Aziz, Natalia Carman Prodan, Zhiqi Yang, Aditya Kumar Lankapalli, Katrin Lazar, Mohamad Nasir Shafiee, Ersoy Kocak, Harivignesh Ganesan, Surya Sekhar Pal, Omer Khalid, Norhana Mohd Kasim, Aida Kalok, Norashikin Abdul Fuad, Alfred Lennart Bissinger, Tina Ganzenmueller, Thomas Iftner, Karl Oliver Kagan, Stephan Ossowski, Nicolas Casadei, Sara Y. Brucker, Olaf Riess, Yogesh Singh, Deutsche COVID-19 OMICS Initiative (DeCOI)

**Affiliations:** ^1^ Department of Women’s Health, Research Institute for Women’s Health, University of Tübingen, Tübingen, Germany; ^2^ Department of Obstetrics and Gynaecology, Faculty of Medicine, Universiti Kebangsaan Malaysia, Kuala Lumpur, Malaysia; ^3^ Department of Biology, Ineos Oxford Institute of Antimicrobial Research, University of Oxford, Oxford, United Kingdom; ^4^ Intitute for Medical Virology and Epidemiology of Viral Diseases, University Hospital Tübingen, Tübingen, Germany; ^5^ Institute of Medical Genetics and Applied Genomics, University of Tübingen, Tübingen, Germany; ^6^ Next Generation Sequencing (NGS) Competence Centre Tübingen (NCCT), University of Tübingen, Tübingen, Germany; ^7^ Department of Obstetrics and Gynaecology, Sungai Buloh Hospital, Selangor, Malaysia

**Keywords:** pregnancy, COVID-19, PBMCs, NK cells, cytotoxic cells, ScRNA-seq, immunophenotyping and cytokines

## Abstract

**Introduction:**

During gestation the immune system undergoes dramatic remodelling to protect the maternal-fetal dyad from infections whilst also preventing fetal rejection. We investigated how SARS-CoV-2 modifies the immune landscape during infection and in recovered pregnant women.

**Methods:**

We immunophenotyped our two independent geographical cohorts using a 14-colour flow cytometry panel (surface and intracellular staining). We estimated cytokines and SARS-CoV-2 IgG antibodies in validation cohort using a multiplexd flow cytometry panel. Single-cell RNA sequencing (scRNA-seq) was performed using a Chromium Single Cell 3’ Gel Bead Chip and Library Kit from 10x Genomics (Drop-seq method). Furthermore, we estimated the cytotoxic functions of natural killer (NK) cells by flow cytometry using surface and intracellular staining.

**Results:**

Using two independent geographical cohorts, we identified that NK cells had a sustained reduction during active infection and after recovery. Further, scRNA-seq data revealed that infection with SARS-CoV-2 rewired the gene expression profile of NK, monocytes, CD4^+^, CD8^+^ effector T cells and antibody producing B cells in convalescent pregnant women. Several gene pathways associated with cytotoxic function, interferon signalling type I & II, and pro- and anti-inflammatory functions in NK and CD8^+^ cytotoxic T cells were attenuated in recovered pregnant patients compared with healthy pregnancies. We validated our scRNA-seq of NK cells from convalescent pregnant women and confirmed that NK cells had diminished levels of cytotoxic proteins; perforin, CD122 and granzyme B.

**Discussion:**

Overall, our study uncovers that SARS-CoV-2 infection deranges the adaptive immune response in pregnant women even after recovery and may contribute to post-COVID19 sequalae of symptoms.

## Graphical Abstract

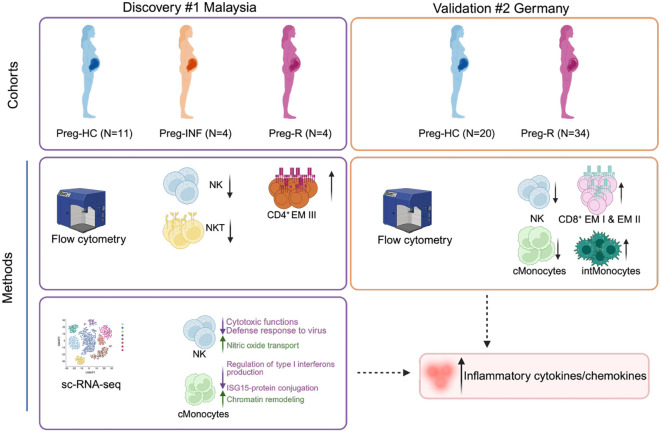

## Introduction

Pregnancy is a complex phenomenon, and the host immune system must maintain an equilibrium to prevent maternal rejection of a semi-allogenic fetus whilst protecting the mother against pathogens (1, 2). Epidemiological evidence from other pandemics (Influenza, Zika, or Ebola virus) has highlighted that pregnant women are more susceptible, develop more severe complications, and are associated with higher adverse pregnancy outcomes (3–7).

Severe Acute Respiratory Syndrome Coronavirus-2 (SARS-CoV-2) is the causative zoonotic pathogen for Coronavirus Disease 2019 (COVID-19), which led to the recent global pandemic in 2019 (8, 9). As of April 2025, more than 7.1 million deaths have been attributed to SARS-CoV-2 infection, and more than 778 million people have been infected (10). Although the pandemic has now subsided, the decreasing protective effects of the current COVID-19 vaccinations coupled with the threat of new infections from new clades (variants) requires that we still need a greater understanding of the pathogenesis and critically, the long-term effects post-infection (11–15). Post-COVID-19 (also known as Long COVID) condition affects more females than males and occurs in approximately 10%–30% of individuals following infection during pregnancy (16). Currently, there are no guidelines for the management of obstetric patients with post-COVID-19 conditions.

The knowledge regarading the immune responses against SARS-CoV-2 infection during pregnancy are conflicting. It has been reported that pregnant women have lower infection rates during the early phase of the pandemic (17). Other studies have reported that pregnant women were mostly asymptomatic (90%) and developed milder symptoms after SARS-CoV-2 infection (18, 19). However, the actual number of infected pregnant women may be higher than that reported because of the limited number of surveillance studies and global shielding programs (20). It has now been corroborated by various groups including ours, that global maternal and fetal outcomes worsened during the COVID-19 pandemic (21–24). Recent data have suggested that age-matched pregnant women are more vulnerable to SARS-CoV-2 infection than healthy nonpregnant women (23). Additionally, SARS-CoV-2 infection in pregnant women leads to increased placental inflammation, predisposition to maternal vascular thrombosis, higher caesarean section rates, fetal growth restriction, preterm birth, preeclampsia and higher rates of hospitalization (24–29). We have also shown that altered villous maturation and severe maternal COVID-19 infection are associated with an elevated risk of poor neonatal Apgar scores and maternal mortality, respectively (24). The general consensus regarding SARS-CoV-2 infection and pregnancy remains obscure, as these comparisons are based on small cohorts, variations in inclusion/exclusion criteria, and different sampling methods/platforms. Furthermore, only a handful of studies have explored how SARS-CoV-2 infection modulates immune cell function during pregnancy (30–34). A recent single-cell RNA sequencing (scRNA-seq) study identified that immunological rewiring may occur at the maternal–fetal interface following asymptomatic or mild SARS-CoV-2 infection; however, the authors did not address whether the immune system returned to normal function in the convalescent phase (35). Another group described a putative link between SARS-CoV-2 infection and T cell exhaustion in pregnancy and they identified a pregnancy-specific gene signature using scRNA-seq, although comparisons were made between non-pregnant and pregnant women (36).

Taken against the backdrop of approximately 778 million reported cases, almost 6 in 100 individuals now suffer from ‘Long COVID’ or post-COVID-19 syndrome (91). Therefore, there is no clear consensus as to whether the response of the immune system is a general phenomenon, and secondly, whether the immune system recovers after SARS-CoV-2 infection during pregnancy. To address these critical gaps, we comprehensively assessed the differences between host immune responses to SARS-CoV-2 infected pregnant women and recovered pregnant women. We used flow cytometry, scRNA-seq, cytokine assessment, and antibody profiling to determine whether SARS-CoV-2 infection could ‘rewire’ the immune response during active infection and ‘reset’ during the recovery phase in pregnant women from two independent geographical cohorts. Our findings highlight the mechanism by which SARS-CoV-2 infection negatively influences the immune response during pregnancy. Our work provides a framework that enables a comprehensive understanding of the impact of SARS-CoV-2 infection during pregnancy on short- and long-term immunity.

## Materials and methods

### Ethical statement and cohort description

Written informed consent was obtained from all invited participants, and the study procedures were carried out in accordance with the Declaration of Helsinki 2000 and the local ethical guidelines. Two geographical cohorts were included in this study. To protect the identities of the enrolled pregnant women, the samples were pseudonymized.

Cohort 1 (discovery): This study was part of the overall study of the transcriptomic and protein analyses of pregnant women with a history of COVID-19 infection at the epicenter of the COVID-19 pandemic in Malaysia, approved by the Research Ethics Committee of the National University of Malaysia (JEP-2021-465) and by the Medical Research Ethics Committee (MREC) of the Ministry of Health Malaysia (ID-58736).

Cohort 2 (validation): Studies involving human participants were reviewed and approved by the Ethics Committee of the Medical Faculty of the Eberhard Karls University Tübingen and the University Hospital Tübingen, Germany (Project No. 671/2023BO1).

### Clinical and sample definitions

Eligibility criteria included age >18 years and either positive RT-PCR test for infection or negative RT-PCR test for healthy controls and/or recovered pregnant women. During the COVID-19 pandemic (April 2020–January 2021), pregnant women visiting the hospital were routinely screened for SARS-CoV-2 infection using an antigen test. Positive results were confirmed using the SARS-CoV-2 RT-PCR test. Samples were prospectively collected from pregnant, unvaccinated women with COVID-19 and were categorized according to National Institutes for Health (NIH) criteria (37, 38). Samples were collected during the period of acute illness (within 30 days of a positive SARS-CoV-2 PCR test) and/or during the convalescent period (>30 days after a positive RT-PCR test). Uninfected contemporaneously pregnant controls (SARS-CoV-2 PCR-negative) were recruited from our prenatal outpatient clinic.

### Demographic description of the cohorts and sample collection

#### Cohort 1 (discovery)

**Figure 1:**
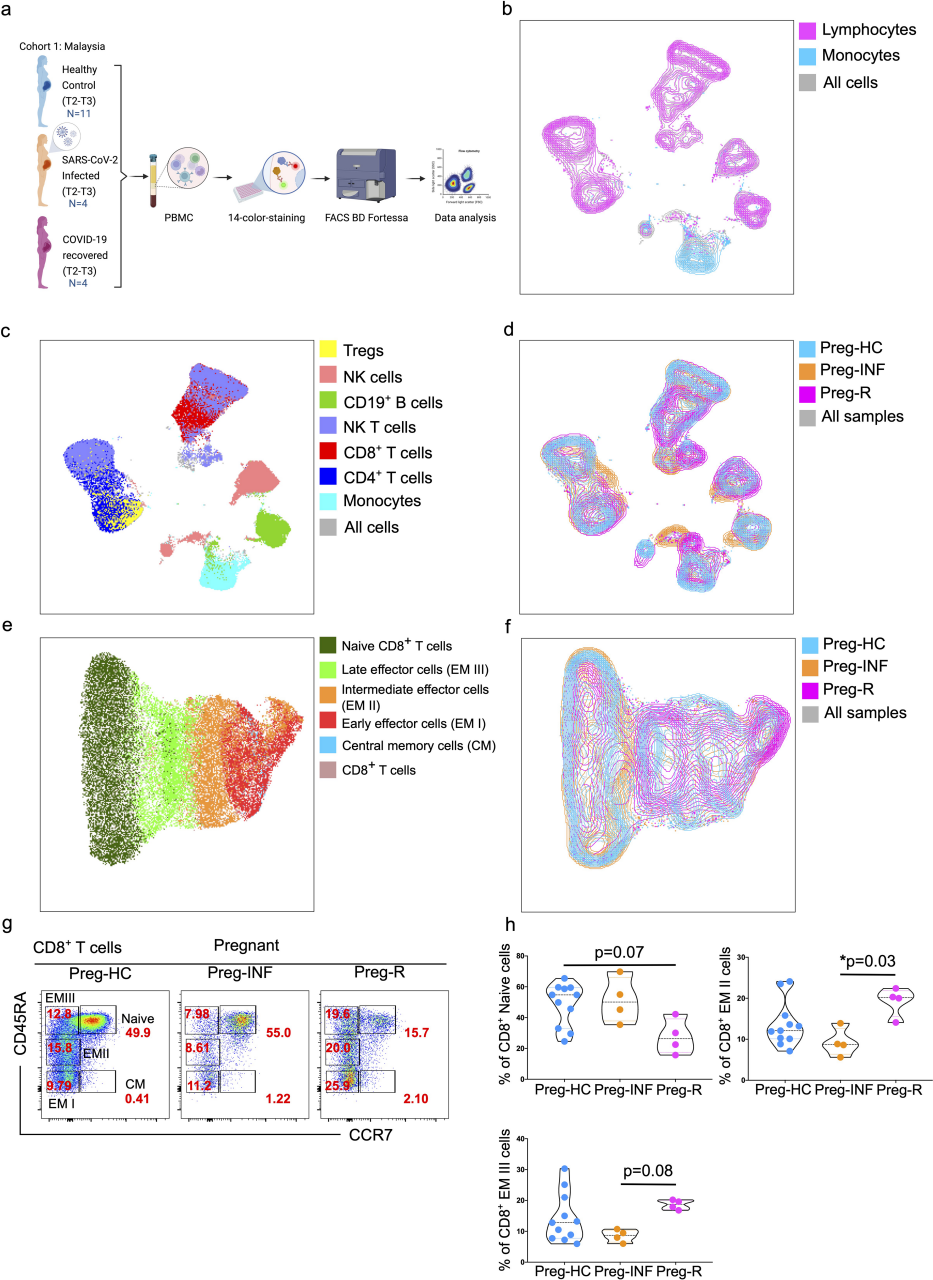
Immunophenotyping of PBMCs from pregnant SARS-CoV-2 infected and recovered patients. **(a)** Malaysian Cohort 1: Description of PBMCs used for immunophenotyping collected from pregnant healthy controls (Preg-HC, blue), SARS-CoV-2 infected (Preg-INF, orange) and COVID-19 recovered (Preg-R, pink). **(b)** Unsupervised clustering of immune cells based on 14-color flow cytometry panel. Each PBMC sample from Preg-HC, Preg-INF, and Preg-R groups were concatenated bioinformatically using the FlowJo software. Two major clusters for lymphocytes (pink) and monocytes (blue) population were identified and presented on a UMAP plot. The combined cell population is shown in gray. **(c)** UMAP plot characterising 7 identified immune subsets; monocytes (cyan), CD4^+^ T cells (navy), CD8^+^ T cells (red), NKT cells (lilac), CD19^+^ B cells (green), NK cells (rose), and Tregs (yellow). **(d)** Overlay of UMAP to show the different immune cell landscape; Preg-HC (blue), Preg-INF (orange), and Preg-R (pink) patient groups. The combined cell population is shown in gray. **(e)** Delineation of all CD8^+^ T subsets. UMAP overlay showing the central memory (blue), early (red), intermediate (orange), late effector (bright green), and Naïve (dark green) CD8^+^ T cells. The combined cell population is shown in rose. **(f)** UMAP overlay depicting the various CD8^+^ T cells based on the different patient groups. Preg-HC (cyan), Preg-R (pink), and Preg-D (orange). The combined cell population is shown in gray. **(g)** Original FACS plots of cytotoxic CD8^+^ T cells. Identification of Naïve, memory, and effector memory CD8^+^ T cells based on CD45RA and CCR7 markers. FACS plots show the CD45RA^+^CCR7^+^ naïve, CD45RA^-^CCR7^+^ CM cells, CD45RA^+high^CCR7^-^ late EM, CD45RA^+mid^CCR7^+^ intermediate EM, CD45RA^-^CCR7^-^ early EM cells in the different patient groups. **(h)** The percentage of Naïve (upper left), intermediate (EM II, right) and late EM III (bottom left) cells are shown as violin plots with each dot represents an individual sample. P-values show the significance among Preg-HC, Preg-INF and Preg-R groups and compared using Kruskal–Wallis test, adjusted for Dunn’s multiple comparisons test. P ≤0.05 considered significant (*p ≤0.05).

Whole blood was collected from pregnant women enrolled at the Department of Obstetrics and Gynecology, Universiti Kebangsaan Malaysia Medical Centre, Kuala Lumpur, Malaysia (cohort 1; discovery cohort). A total of 19 pregnant Malaysian and Malaysian Indian pregnant women were recruited. The cohort consisted of pregnant healthy controls (Preg-HC; N = 11), pregnant SARS-CoV-2 infected (Preg-INF; N = 4) and recovered from SARS-CoV-2 infection (Preg-R; N = 4; **Figure 1a**, **Supplementary Table 1**). Some of these patients were described in our previous publication (39). Infected pregnant women were either asymptomatic (N = 3) or had mild/moderate COVID-19 manifestations (N = 1). No severe forms of COVID-19 patients were reported and thus were not included in this study. The recovered pregnant women were initially asymptomatic (N = 2) or had mild/moderate manifestations of COVID-19 (N = 2). The study participants were of similar age and trimester; healthy pregnant controls (32 years ± 2.93, 31.4 weeks ± 9.5), infected pregnant (32 years ± 6.97, 33.5 weeks ± 7.14), and recovered pregnant women (32 years ± 5.22, 25 weeks ± 9.67) (**Supplementary Table 1**).

Blood was collected from pregnant women infected with SARS-CoV-2 1–3 days after hospital admittance or prior caesarean section delivery. Pregnant women who recovered from SARS-CoV-2 were admitted prior to delivery, and for pregnant women from the healthy control group who attended the outpatient clinic, 3 mL–5 mL of blood was collected in 5 mL or 9 mL lithium heparin vacutainer tubes. The serum was isolated by centrifugation immediately after collection. Human Peripheral Blood Mononuclear Cells (PBMCs) were isolated by the Ficoll method, as described previously, and PBMCs were stored in DMSO at −80°C (40).

#### Cohort 2 (validation)

For the second cohort, pregnant women seropositive for COVID-19 IgG antibodies were retrospectively categorized. The German cohort was used to validate the data obtained from Cohort 1. Healthy controls (N = 20; 31 years ± 4.96, 23.5 weeks ± 9.28) and pregnant (N = 34; collection time point 1 (CT1)—32.79 years ± 4.96, 17.1 weeks ± 7.06, CT2—33.25 years ± 4.82, 22.3 weeks ± 7.33, and CT3—33.57 years ± 5.23, 26.7 weeks ± 9.85) women recovered from SARS-CoV-2 infection (**Supplementary Table 2**) were enrolled. All samples were used for cytokine and SARS-CoV-2 antibody data analysis. For immunophenotyping, PBMCs were collected from healthy controls (N = 20; 31 years ± 4.96, 23.5 weeks ± 9.28) and pregnant (N = 14; 32.42 years ± 4.94, 21.3 weeks ± 9.72) women recovered from SARS-CoV-2 infection (**Supplementary Table 2**). Healthy control pregnant women who had no SARS-CoV-2 vaccination or infection (based on a negative SARS-CoV-2 antigen test and baseline or reduced Nucleocapsid IgG antibody levels) in the last 6 months based on a patient questionnaire. Sample collection was performed at the University Hospital for Women, Tübingen, Germany (cohort 2; validation).

### Preparation of PBMCs for scRNA-seq and antibody (surface and intracellular proteins) staining for flow cytometry

Frozen PBMCs were thawed at 37°C in a water bath for 2 min, or until a few small ice crystals remained. In a biosafety hood (Level 2), thawed cells were transferred to a 50 mL conical tube. The original cryovial was rinsed with 1 mL of RPMI1640 complete medium (RPMI1640, 10% FBS, and 1% antibiotic/antimycotic) to remove any residual PBMCs. Next, warmed RPMI1640 complete medium was added dropwise using a Pasteur pipette while gently vortexing the tube up to a volume of 32 mL. Subsequently, the Falcon tube was centrifuged at 400×*g* for 5 min at room temperature. The supernatant was discarded, leaving 1 mL of residual medium, and the cell pellet was resuspended using a 10 mL Pasteur pipette. Following this, 19 mL of 10% RPMI1640 complete medium was added and then centrifuged at 400×*g* for 5 min at room temperature to remove any remaining DMSO. The supernatant was discarded, the cell pellet resuspended in 2 mL of medium, and the cells were counted using an automated Bio-Rad cell counter. Approximately, 1.0 × 10^6^ live cells were used of the 14 color FACS panel and 0.5 × 10^6^ cells used for scRNA-Seq.

### Flowcytometry immunophenotyping (14-color) staining and data analysis

For flow cytometric staining, 1 × 10^6^ cells were added to a 96-well plate and washed with DPBS (Ca^2+/^Mg^2+^ free from DPBS; # D8537, Sigma, Germany). First, the cells were stained with 5 µL of live and dead dye (1:400 dilution in PBS; #L34993, LIVE/DEAD™ Fixable Near IR (780) Viability Kit, for 633 nm excitation, Thermofisher, Germany) and incubated for 15 min in the dark at room temperature. After incubation, 195 µL DPBS was added to the cells and centrifuged at 400×*g* for 5 min at room temperature. After washing the cells, the supernatant was discarded and 50 µL of DPBS was added to each well. A 12-color antibody cocktail was used to identify cell-surface markers: CD3, CD4, CD8, CD19, CD56, HLA-DR, CD38, CD154, CD45RA, CCR7, CD14, and CD16, as reported previously (40). For each reaction, we used 2.5 µL of each antibody together with 5 µL of superbright staining buffer (to distinguish two different antibodies in the superbright channel) per sample. The cells were incubated with the antibody cocktail for 30–40 min at room temperature. After incubation, the cells were washed with 100 µL of DPBS and fixed using 100 µL of Fix/Permb buffer (Thermo Fisher, Germany) for 45 min. After fixation, the cells were permeabilized using 100 µL of 1× permeabilization buffer. After the fixation and washing steps, 2 µL of FOXP3 intracellular antibody was added and incubated at room temperature for 30–40 min. After incubation, the cells were washed with 150 µL of PBS, and cell samples were acquired on a BD LSRFortessa Cell Analyzer. The data were analyzed using FlowJo for 2D FACS plot dimensional reduction methods. Overall, 100,000–200,000 cells were acquired from each sample and live cells were used for further analysis. The gating strategy for live lymphocytes and monocytes is shown in **Supplementary Figure 1**.

### 13-plex Human inflammation panel I cytokine and chemokine detection

Serum samples were tested for various cytokines, including IL-1β, IFN-α2, IFN-γ, TNF-α, MCP-1 (CCL2), IL-6, IL-8 (CXCL8), IL-10, IL-12p70, IL-17A, IL-18, IL-23, and IL-33, using a bead-based assay kit, the LEGENDplex™ Human Inflammation Panel-1 (#740809; BioLegend, USA). All the reagents adjusted to room temperature prior to use. First, standard concentrations were prepared by reconstituting the lyophilized cocktail with 250 µL of LEGENDplex™ Assay Buffer to obtain the highest concentration (C7). Serial 1:4 dilutions were performed to obtain standards C6 to C1, with the LEGENDplex™ Assay Buffer used as the 0 pg/ml standard (C0), and the LEGENDplex™ Assay was performed in a V-bottom plate. To each well, 12.5 µL of standards or plasma samples were added to each well, followed by LEGENDplex™ Matrix-B3 Solution in standard wells or Assay Buffer in sample wells. After vortexing, 12.5 µL of pre-mixed beads were added to each well. The plate was sealed, wrapped in aluminum foil to prevent photobleaching, and incubated at room temperature on a shaker at 800 rpm for 2 h. After incubation, the plate was centrifuged at 250×*g* for 5 min using a swinging bucket rotor and the supernatant was removed by flicking the plate. The beads were washed with LEGENDplex™ Wash Buffer, centrifuged and the flow-through was discarded. The excess buffer was removed using a paper towel. Next, 12.5 µL of the detection antibody mixture was added to each well. The plate was then sealed and placed on a shaker at 800 rpm for an hour. Without washing, 12.5 µL SA-PE buffer was added to each well and incubated on a shaker at 800 rpm for an additional 30 min. The plate was centrifuged again at 250×*g* for 5 min, and the supernatant was removed. Centrifugation was performed twice to reduce background noise. The beads were resuspended by adding 150 µL of LEGENDplex™ Wash Buffer (1×) to each well. The flow cytometer (BD LSRFortessa™ Cell Analyzer) settings and compensation were initially set up using a 2-color with single antibody-stained ‘control’ beads and with a negative control. Once the compensation matrix was confirmed, samples were acquired. Prior to inserting into the FACS tube, the samples were vortexed for 5 s to prevent bead clumping. The flow rate was set to medium and the number of beads acquired was set to 10,000 events per sample. Data were analyzed using BioLegend’s LEGENDplex™ Data Analysis Software. The concentrations of cytokines and chemokines are shown in the figures in pg/mL.

### 3-Plex SARS-CoV-2 serological IgG antibodies detection

IgG antibodies against SARS-CoV-2 Spike Protein S1, Nucleocapsid and Spike Protein RBD were quantified in serum samples collected from recovered (Preg-R) and healthy control pregnant women (Preg-HC) using a bead-based assay kit (LEGENDplex™ SARS-CoV-2 Serological IgG Panel; #741132; BioLegend, USA). Standard concentrations were prepared by reconstituting the lyophilized cocktail with 250 µl of the LEGENDplex™ Assay Buffer. From the highest concentration (C7), serial 1:4 dilutions were performed to obtain standards C6 to C1, with the LEGENDplex™ Assay Buffer used as the 0 pg/ml standard (C0). Serum samples were diluted (1:100) with LEGENDplex™ Assay Buffer. 25 µL of standards and plasma/serum samples was added to each well of a V-bottom plate, followed by 25 µL of LEGENDplex™ Assay Buffer and 25 µL of the pre-mixed beads. The plate was sealed and covered with aluminum foil to prevent photo bleaching. The pre-mixed bead bottle was vortexed before being adding to the assay wells. The plate was then placed in a shaker at 800 rpm for 2 h. After incubation, the plate was centrifuged at 250×*g* for 5 min using a swinging bucket rotor. The supernatant was carefully removed by gentle flicking of the plate under continuous motion. The centrifugation step was repeated after washing the beads with LEGENDplex™ Wash Buffer. The plate was then dried using tissue towels and 12.5 µL of detection antibodies mixture was added to each well. The plate was then sealed and placed on a shaker at 800 rpm for an hour. 25 µL SA-PE buffer was added to each well and incubated on a shaker at 800 rpm for 30 min. The plate was centrifuged again at 250×*g* for 5 min and the supernatant removed as before. The centrifugation step was performed twice to reduce the background noise. The beads were resuspended by adding 100 µL of LEGENDplex™ Wash Buffer (1×) to each well. The flow cytometer (BD LSRFortessa™ Cell Analyzer) settings and compensation were initially set up using a 2-color with single antibody-stained ‘control’ beads and with a negative control. Once the compensation matrix was confirmed the samples were then acquired. Prior to inserting the FACS tube, the samples were vortexed for 5 s to prevent bead clumping. The flow rate was set to medium and the number of beads acquired was set to 5,000 events per sample. Data were analyzed using BioLegend’s LEGENDplex™ Data Analysis Software. The concentration of IgG presented in the figures was µg/mL.

### Sample preparation for single cell RNA sequencing

Thawed 0.5 × 10^6^ PBMCs were washed three times at 400×*g* for 5 min at room temperature with 1× staining buffer (BioLegend) and kept on ice. Live dead staining using acridine orange and propidium iodide dye (#TN184; DeNovix, USA) was performed to measure the (live) cells before proceeding to 10× Genomics experiments (41). We followed the 10× chromium sc-RNA-seq protocol as described previously (42).

PBMCs were separated into single cells and prepared using the Chromium Single Cell Gene Expression Solution, Chromium Single Cell 3’ Gel Bead, Chip, and Library Kits v1 (10× Genomics) according to the manufacturer’s protocol. In brief, 20,000 cells were loaded into each channel with an average expected recovery of 6,000–9,000 cells (approximately 40%–50% recovery). The cells were then partitioned into Gel Beads in Emulsion in the Chromium instrument, where cell lysis and barcoded reverse transcription of mRNA occurred, followed by amplification, shearing, and 3’ adapter and sample index attachment. Libraries were quantified by a Qubit™ 2.0 Fluorometer (Thermo Fisher) and fragment size was controlled using a 2100 Bioanalyzer with High Sensitivity DNA kit (Agilent). Sequencing was performed in paired-end mode with a S2 flow cell (100 cycles) using NovaSeq 6000 sequencer (Illumina, USA) at the NCCT Core facility, Tübingen, Germany.

For the 10× Genomics sequencing data alignment and quantification, the sequencing data were processed using CellRanger software (v3.0.1) with default parameters and the GRCh38 v3.0.0 human reference genome. The output file-filtered feature matrix files were used for further analyses.

### scRNA-seq analysis by Seurat pipeline

First, the cell ranger-filtered feature matrix files were converted into individual Seurat obj files. We used eight samples: N = 4 Preg-HC and N = 2 Preg-R (run in duplicate) samples for analysis (total cells recovered from sequencing; n = 55,588). We used the basic filter criteria min.cells = 3, min.features = 200 for making individual Seurat Objects that left the remaining 46,594 cells in a mergeSeuratObject (mergeGH). Later, filtering steps were performed to keep the nCount_RNA (>800 and <10,000), nFeature_RNA (>200 and >5,000), and mitoPercent (<20) in a filtered SeuratObject (mergeGH_filtered).

After filtering 30,394 high-quality single cells, we performed cell clustering and gene expression analysis after integrating data from all samples. We performed the standard Seurat workflow (normalize data, find variable features, scale data, run PCA, find neighbors, find clusters, and run UMAP) to identify any batch effects before integration of all data using the canonical correlation analysis (CCA) integration method to obtain the final Seurat object (obj). This obj has been used to plot the UMAP figures in the manuscript. We identified cell markers for individuals cell clusters using “FindAllMarkers” function from Seurat and based on gene expression to classify into different cell clusters/cell types.

T cells (CD3E) were classified into 14 subclasses, including naïve CD4 T cell (CD4 and CCR7), CD4 T effector memory (CD4 TEM, CD4, TRAC, and FYB1), CD4 T effector memory RA (CD8 TEMRA, ANK3, and CD28), CD4 central memory T cell (CD4 TCM, MAL, FHIT, and LEF1), CD4 cytotoxic T lymphocyte cell (CD4 CTL, ITGB1, GZMA, GZMB, B3M, IL32, KLRB1, and KLRG1), Treg (FOXP3 and TIGIT), naïve CD8 T cell (CD8A and CCR7), CD8 effector memory (CD8 TEM, CD8A, and GZMA), CD8 effector memory RA (CD8 TEMRA, ANAX1, TGFBR3, and GZMH), CD8 central memory T cells (CD8 TCM, GZMK, and YBX3), MAIT (TRAV1-2), γδ T cells (TRGV9 and TRDV2), proliferative T lymphocytes (MKI67), and NKT cells (NKG7, CD3E). B cells (CD79A) were classified as naïve B cells (MS4A1, CCR7, and SELL), memory B cells (MS4A1), and plasmablasts (MZB1 and IL3RA). Innate immune cells were classified into classical monocytes (LYZ, CD14, and FCGR3A^−^), plasmacytoid monocyte (LYZ and FCGR3A), and NK cells (GNLY and NKG7).

### Differential gene expression in Preg-HC vs Preg-R for different cell subsets

Each cell cluster was compared for differential expressed genes (DEGs) within the same cell type across two conditions—Preg-R vs Preg-HC to identify the upregulated and downregulated gene expression for gene enrichment analysis based on Seurat function “FindMarker” function. Volcano plots were used to identify differentially regulated genes to ascertain genes expressed above a certain fold change (log_2_FC cutoff = 0.5) and statistically significant adjusted p-values (p ≤0.05). By default, p-values were adjusted using the False Discovery Rate (FDR) and calculated according to the Benjamini-Hochberg algorithm and considered significant (p ≤0.05). Selected highly upregulated and downregulated genes are shown in volcano plots.

### Gene set enrichment analysis

Gene Set Enrichment Analysis (GSEA) was used to determine whether a pre-defined set of genes (p ≤0.05) in a particular subset of cells (e.g., those belonging to a specific Gene Ontologoy (GO) term or Kyoto Encyclopedia of Genes and Genomes (KEGG) pathway) shows statistically significant, concordant differences between Preg-R and Preg-HC groups. We used “Clusterprofiler v3.20,” pathview, enrichplot, wordcloud, msigdbr R packages for data analysis. Gene set enrichment analysis was performed using the gseGO function, keeping the default values. “DOSE” R-package was used for making dotplots for gene set enrichment (gse) or KEGG data for top 10 pathways followed by GSEA plots for the selected genesetIDs.

### Estimation of cytotoxic functions of NK cells at native state using flow cytometry

To understand the cytotoxic functions of NK cells in the native state, 1–2 × 10^6^ PBMCs were taken in a 96 well plate and washed with DPBS (Ca^2+/^Mg^2+^ free). PBMCs were washed again with DPBS, 50 µL of DPBS was added to each well and 2.5 µL Fc-blocking reagent was added, and PBMCs were incubated for 15 min at room temperature. After incubation, the PBMCs were then stained with two-color antibody cocktail for surface recognition markers (CD8 and CD56). For each reaction, we used 2.5 µL of antibody. The cells were then incubated with an antibody cocktail for 30–40 min. After incubation, the cells were washed with PBS and fixed using fix/permeabilization buffer for 45 min. After fixation, the cells were permeabilized using 1× permeabilization buffer and added 2 µL of perforin, granzyme B, IL-10, and IFN-γ intracellular antibodies and incubated at room temperature for 30–40 min. After incubation, cells were washed with PBS, and cell samples were acquired using BD Fortessa flow cytometry and then analyzed using FlowJo software.

To test the expression of CD122 and CD215 on NK cells and monocytes, we stained PBMCs with CD3, CD4, CD8, CD19, CD56, CD122, CD215, CD14, and CD16 (each with 2.5 µL) antibodies and incubated for 30–40 min. After incubation, the cells were washed with PBS and fixed using fix/permeabilization buffer for 45 min. After incubation, the cells were washed with PBS, and cell samples were acquired using BD Fortessa flow cytometry. Data were analyzed using FlowJo software.

### Statistics analysis and data visualization

Cytometric data were analyzed using FlowJo 10.8.1. Figures prepared using ggplot2 in R package V4.4.2 for cytokines, flow cytometry immunophenotyping (Cohort 2), and final figures assembled in Inkscape (https://inkscape.org) for the manuscript. Statistical details of the experiments are provided in the respective figure legends. No matching or pairing of the samples was used in the statistical analysis. Statistical analyses were performed using GraphPad Prism 10.0, unless otherwise stated. Statistical analysis was conducted without the assumption of normally distributed data; therefore, non-parametric tests Kruskal-Wallis and Wilcoxon rank-sum tests were used. Nonparametric test—Kruskal–Wallis test with Dunn’s *post-hoc* test (for multiple comparisons) was applied for unpaired comparisons using Prism 10.0 (Cohort 1). To compare two groups of nonparametric interval or non-normally distributed data, the Wilcoxon rank-sum test was used using R package 4.4.2 (Cohort 2). Results with p values of up to 0.09 were not considered statistically significantly different but were discussed in the text as indicative of a difference between groups. Furthermore, an experimental plan drawing was made using BioRender.com. A table of key resources is provided for all the reagents and tools used in this manuscript (**Table 1**).

**Table 1:** Key resources table.

Reagent/resource	Reference or source	Identifier or catalog number
Experimental models		
PBMCs Cohort 1	This study	N/A
PBMCs Cohort 2	This study	N/A
Antibodies		
Anti-Human CD3 eFlour™ 450 (clone UCHT1)	Thermo Fisher Scientific (eBioscience™)	Cat#48-0038-42
Anti-Human CD4 Super Bright™ 600 (clone SK-3)	Thermo Fisher Scientific (eBioscience™)	Cat#63-0047-42
Anti-Human CD8a PerCP-eFlour™ 710 (clone SK1)	Thermo Fisher Scientific (eBioscience™)	Cat#46-0087-42
Anti-Human CD19 eFlour™ 506 (clone HIB19)	Thermo Fisher Scientific (eBioscience™)	Cat#69-0199-42
Anti-Human CD56 PE (clone MEM-188)	Thermo Fisher Scientific (eBioscience™)	Cat#MA1-19638
Anti-Human CD45RA PE-Cyanine7 (clone HI100)	Thermo Fisher Scientific (eBioscience™)	Cat#25-0458-42
Anti-Human HLA-DR Alexa Fluor™ 647 (clone L243)	Thermo Fisher Scientific (Invitrogen)	Cat#A51010
Anti-Human CD38 PE-eFlour™ 610 (clone HIT2)	Thermo Fisher Scientific (eBioscience™)	Cat#61-0389-42
Anti-Human CD154 (CD40 Ligand) PE-Cyanine5 (clone 24-31)	Thermo Fisher Scientific (eBioscience™)	Cat#15-1548-42
Anti-Human CD197 (CCR7) Brillian Violet 785™ (clone G043H7)	BioLegend	Cat#353230
Anti-Human CD14 Alexa Fluor™ 700 (clone 61D3)	Thermo Fisher Scientific (eBioscience™)	Cat#56-0149-42
Anti-Human CD16 Super Bright™ 702 (clone 3G8)	Thermo Fisher Scientific (eBioscience™)	Cat#67-0166-42
Anti-Human CD69 APC-eFluor™ 780	Thermo Fisher Scientific (eBioscience™)	Cat#47-0699-42
Anti-Human FOXP3 FITC (clone PCH101)	Thermo Fisher Scientific (eBioscience™)	Cat#11-4776-42
LIVE/DEAD™ Fixable Near-IR Dead Cell Stain Kit, for 633 or 635 nm excitation	Thermo Fisher Scientific (Invitrogen)	Cat#L10119
Anti-Human IFN-γ Alexa Fluor™ 488 (clone 4S.B3)	Thermo Fisher Scientific (eBioscience™)	Cat#53-7319-42
Anti-Human Granzyme B Alexa Fluor™ 488 (clone 351927)	Thermo Fisher Scientific	Cat#MA5-23639
Anti-Human Perforin PE-Cyanine7 (clone delta G9 or dG9)	Thermo Fisher Scientific (eBioscience™)	Cat#25-9994-42
Anti-Human IL-10 PE-Cyanine 7 (clone JES3-97D)	Thermo Fisher Scientific (eBioscience™)	Cat#25-7108-42
Anti-Human Granzyme A PE (clone CB9)	Thermo Fisher Scientific (eBioscience™)	Cat#12-9177-42
Anti-Human IL-17 PE-eFluor™ 610 (clone eBio64DEC17)	Thermo Fisher Scientific (eBioscience™)	Cat# 61-7179-42
Anti-Human TNF-α eFluor ™ 450 (clone MAb11)	Thermo Fisher Scientific (eBioscience™)	Cat# 48-7349-42
Anti-Human CD122 (IL-2Rβ) Alexa Flour™ 647 (clone TU27)	BioLegend	Cat#339008
Anti-Human CD215 PE-Cyanine 7	BioLegend	Cat#
eBioscience™ Foxp3/Transcription Factor Staining Buffer Set	Thermo Fisher Scientific (eBioscience™)	Cat#00-5523-00
Chemicals and other reagents		
RPMI1640	Thermo Fisher	Cat# 61870036
Antibiotics/Antimycotic	Thermo Fisher	Cat# 15240062
FBS	Thermo Fisher	Cat# A5256701
Ficoll Hypaque	PAN BIOTECH	Cat# P04-601000
LEGENDplex™ Human Inflammation Panel 1 (13-plex) with V-bottom Plate	BioLegend	Cat#740809
LEGENDplex™ SARS-CoV-2 Serological IgG Panel (3-plex) w/VbP	BioLegend	Cat#741132
Cell Staining Buffer	BioLegend	Cat#420201
N 184 DeNovix Acridine Orange/Propidium Iodide Assay	DeNovix	Cat#TN184
Trypan Blue	Sigma-Aldrich	Cat#T8154-100ML
High Sensitivity DNA kit	Thermo Fisher Scientific	Cat# Q33230
S1 and S2 flow cell (100 cycle)	Illumina, USA	Cat#
Super Bright Complete Staining Buffer	Thermo Fisher Scientific (eBioscience™)	Cat#SB-4401-42
UltraComp eBeads™ Compensation Beads	Thermo Fisher Scientific (Invitrogen)	Cat# 01-2222-42
Dynabeads™ MyOne™ SILANE	10× Genomics	Cat#PN-2000048
Chromium Single cell 3’ GEM Library & Gel Bead Kit v3, 16rxns	10× Genomics	Cat#PN-1000075
Chip B	10× Genomics	Cat#PN-1000074
Chromium i7 Multiplex Kit, 96 rxns	10× Genomics	Cat#PN-120262
Software		
Inkscape v1.2.2	Inkscape	https://inkscape.org/release/inkscape-1.2/
GraphPad Prism 10.0	Graphpad	https://www.graphpad.com
R version V4.4.0		https://cran.r-project.org/bin/windows/base/old/
R Studio Version 2024.09.1 + 394 (2024.09.1 + 394)		https://posit.co/download/rstudio-desktop/
Microsoft^®^ Excel for Mac Version 16.96 (25041326)		Microsoft 365 Subscription
fgsea v1.30.0		https://bioconductor.org/packages/release/bioc/html/fgsea.html
Metascape		https://metascape.org/gp/index.html#/main/step1
clusterprofiler v4.12.6		https://bioconductor.org/packages/release/bioc/html/clusterProfiler.html
EnhanceVolcano v1.22.0		https://bioconductor.org/packages/release/bioc/html/EnhancedVolcano.html
Flow jo 10.10	Flowjo	https://www.flowjo.com/flowjo/download
CellRanger software v3.0.1	10× Genomics	https://github.com/10XGenomics/cellranger
Human reference genome GRCh38 v3.0.0		https://www.ncbi.nlm.nih.gov/datasets/genome/GCF_000001405.26/
Seurat 5.2.0		https://satijalab.org/seurat/
ggplot2 3.5.3		https://ggplot2.tidyverse.org
Tidyverse v2.0.0		https://www.tidyverse.org
gridExtra v2.3		https://cran.r-project.org/web/packages/gridExtra/index.html
SeuratWrappers v0.3.5		https://github.com/satijalab/seurat-wrappers
presto		https://github.com/immunogenomics/presto
dplyr		https://dplyr.tidyverse.org
patchwork		https://cran.r-project.org/web/packages/patchwork/index.html
cowplot v		https://cran.r-project.org/web/packages/cowplot/vignettes/introduction.html
SeuratData v0.2.2.9001		https://github.com/satijalab/seurat-data
scales v1.3.0		https://scales.r-lib.org
reshape2 v1.4.4		https://cran.r-project.org/web/packages/reshape2/index.html
Azimuth		https://github.com/satijalab/azimuth
scattermore v1.2		https://cran.r-project.org/web/packages/scattermore/index.html
hdf5r v1.3.12		https://cran.r-project.org/web/packages/hdf5r/index.html
ComplexHeatmap v2.20.0		https://bioconductor.org/packages/release/bioc/html/ComplexHeatmap.html
dittoseq v1.16.0		https://bioconductor.org/packages/release/bioc/html/dittoSeq.html
viridis v0.6.5		https://cran.r-project.org/web/packages/viridis/vignettes/intro-to-viridis.html
pathview v1.44.0		https://www.bioconductor.org/packages/release/bioc/html/pathview.html
enrichplot v1.24.4		https://bioconductor.org/packages/release/bioc/html/enrichplot.html
wordcloud v2.6		https://cran.r-project.org/web/packages/wordcloud/index.html
msigdbr v10.0.1		https://cran.r-project.org/web/packages/msigdbr/index.html
DOSE v3.30.5		https://www.bioconductor.org/packages/devel/bioc/vignettes/DOSE/inst/doc/DOSE.html
UpSetR v1.4.0		https://cran.r-project.org/web/packages/UpSetR/index.html
Parallel v4.4.2		https://cran.r-project.org
ggpubr v0.6.0		https://cran.r-project.org/web/packages/ggpubr/index.html
FSA v0.9.6		https://cran.r-project.org/web/packages/FSA/index.html
Rstatix v0.7.2		https://cran.r-project.org/web/packages/rstatix/index.html
Deposited data		
scRNA-seq data	This study	Zenodo accession ID: https://doi.org/10.5281/zenodo.14066080
Code used for single cell RNA sequencing analysis and figures generation	This study	https://github.com/ysinghbt/scPREG-R

## Results

### Early and late effector CD4^+^ or CD8^+^ T cells were dysregulated in Preg-INF and Preg-R

To decipher the critical immunological drivers that shape host immune responses in pregnant women infected with SARS-CoV-2, we utilized a 14-color antibody panel to profile the immune cell composition with flow cytometry using three patient groups (**Figure 1a**). We first explored the percentage of different immune cells in PBMCs to evaluate the distribution of innate and adaptive immune cell subsets in healthy pregnant women, those infected with SARS-CoV-2 or recovered. The gating strategy for live lymphocytes and monocytes is shown in the FACS plots (**Supplementary Figures 1A, B**). The total fraction of lymphocytes was significantly (p = 0.02) reduced in the Preg-R group compared to that in Preg-HC group (**Supplementary Figures 1B, C**). We observed a significantly (p = 0.03) reduced fraction of monocytes in Preg-INF compared with Preg-HC, while a diminished tendency of monocytes in Preg-R patients compared with Preg-HC (**Supplementary Figures 1B, D**). No apparent difference was observed in either CD4^+^ or CD8^+^ T cells among the Preg-INF, Preg-R, and Preg-HC groups (**Supplementary Figures 1E–G**). However, there was a tendency for decreased CD8^+^ T cells and increased CD4^+^ T cells in Preg-INF compared to Preg-R or Preg-HC.

Next, we examined different subtypes of T, B, NK, NKT, and Treg cells (**Figure 1**). Gated live cells from all 19 samples were concatenated and subjected to unsupervised clustering analysis using uniform manifold projection and approximation (UMAP) to classify the clustering of immune cells and identify differences in immune cell subsets, particularly monocytes and lymphocytes (**Figure 1b**). We observed seven major clusters of cells based on 14-color flow parameters, including monocytes, CD4^+^ T cells, CD8^+^ T cells, CD19^+^ B cells, NKT cells, CD56^+^ NK cells, and Treg subsets (**Figure 1c**). The UMAP analysis was further extended based on individual patients and control groups for the visual inspection of different immune cell subsets (**Figure 1d**). Furthermore, using supervised clustering of CD8^+^ T cells, we identified five different subsets of CD8^+^ T cells, including naïve, central memory (CM), and three different effector memory (EM I–III) subtypes, using sub-UMAP analysis (**Figure 1e**). These CD8^+^ T cell subsets were partitioned into their respective patient groups (**Figure 1f**). Different subsets of CD8^+^ T cells are shown in the original FACS plots of the individual groups for their activation (CD45RA) and naïve (CCR7) markers (**Figure 1g**). We found that naïve CD8^+^ T cells were reduced in Preg-R patients compared with the Preg-HC group; however, this difference was not statiscally significant (p = 0.07) (**Figure 1h**; upper left plot). Intermediate effector CD8^+^ T (EM II) cells were significantly reduced in Preg-INF compared to Preg-R (p = 0.03) (**Figure 1h**; upper right plot). Late effector CD8^+^ T (EM III) or T effector memory re-activated (TEMRA) cells were also reduced in Preg-INF compared to Preg-R (p = 0.08), although the difference was not significant (**Figure 1h**; bottom left plot).

**Figure 2:**
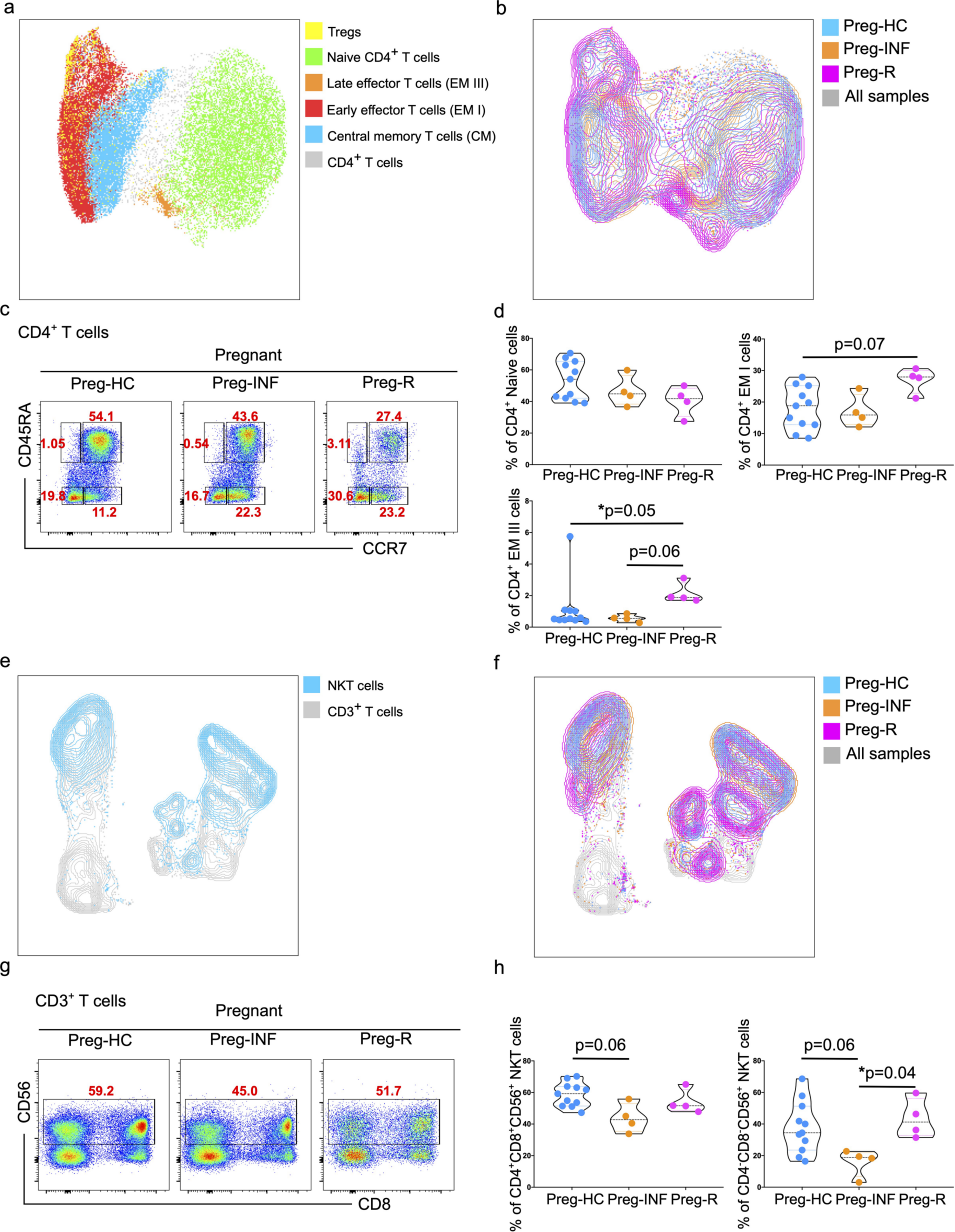
Increased effector CD4^+^ T cells and NKT cells in infected and recovered pregnant women. **(a)** UMAP analysis of total CD4^+^ T cells into different CD4^+^ T subsets including Naïve T cells (green), central memory T cells (CM; blue), early effector T cells (EMI; red), late effector cells (EMII; orange), and Tregs (yellow). The combined cell population is shown in gray. **(b)** UMAP for different CD4^+^ T subsets showing Preg-HC (cyan), Preg-R (pink), and Preg-INF (orange). The combined cell population is shown in gray. **(c)** Original FACS plots identifies CD4^+^ T cells subsets based on CCR7 and CD45RA markers. Classification of naïve, memory and effector memory CD4^+^ T cells based on CD45RA and CCR7 markers. FACS plots show the CD45RA^+^CCR7^+^ Naïve, CD45RA^-^CCR7^+^ CM cells, CD45RA^+high^CCR7^-^ late EM, CD45RA^+mid^CCR7^+^ intermediate EM, CD45RA^-^CCR7^-^ early EM cells. **(d)** The percentage of Naïve, early, and late EM cells are shown in violin plots. P-values show the significance among Preg-HC, Preg-INF, and Preg-R groups and compared using Kruskal–Wallis test, adjusted for Dunn’s multiple comparisons test. P ≤0.05 considered significant (*p ≤0.05). **(e)** UMAP plots showing the distribution of NKT cells (blue) in the CD3^+^ T cell compartment. **(f)** UMAP displaying the overlay with different patient groups. Preg-HC (cyan), Preg-R (pink), and Preg-INF (orange) for NKT cells. Combined samples are shown in gray. **(g)** Original FACS plots presenting the expression of CD56 on CD3^+^CD8^+^ T cells with antibody markers staining for CD8 and CD56. **(h)** The percentage of NKT cells displayed by violin plots among Preg-HC, Preg-INF, and Preg-R groups. Kruskal–Wallis test, adjusted for Dunn’s multiple comparisons test was used to determine significance (*p ≤0.05).

Next, CD4^+^ T cells were sub-grouped and subjected to unsupervised sub-UMAP clustering analysis. We identified four different subsets of CD4^+^ T cells (central memory, early effector, late effector, and naïve T cells) and Tregs, and visualized them according to their respective groups (**Figures 2a, b**). We observed that naïve CD4^+^ T cells tended to be lower in Preg-INF and Preg-R compared with Preg-HC (**Figures 2c, d**, upper right). Furthermore, based on CD45RA and CCR7 markers, we found that early effector (EM I) CD4^+^ T cells tended to increase in Preg-R compared with Preg-HC (p = 0.07); however, this did not reach a significant level (**Figures 2c, d**; upper left plot). However, late effector cells (EM III) were significantly increased (p = 0.05) in Preg-R compared with those in Preg-HC (**Figures 2c, d**; bottom left plot). Overall, it appears that naïve T cells were reduced, while intermediate or later effector memory T cells were increased in Preg-R compared to HC.

### Decreased CD3^+^CD4^-^CD8^-^ NKT and NK cells in Preg-INF or Preg-R

UMAP analysis was performed to determine the distribution of lymphoid natural killer T (NKT) cells. A clear distinction of these cells was identified in the different groups, with the Preg-R group clustering towards the bottom (**Figures 2e, f**). Furthermore, a reduced frequency of CD3^+^CD4^+^CD8^+^CD56^+^ NKT cells was observed in Preg-INF women compared to Preg-HC (**Figures 2g, h**; FACS plots and left violin plot). In addition, we examined the CD3^+^CD4^-^CD8^-^CD56^+^ NKT cell population. We identified a significant reduction (p = 0.04) in Preg-INF compared to Preg-R (**Figures 2g, h**; FACS plot and right violin plot).

Subsequently, myeloid CD3^-^CD56^+^ NK cells were characterized using flow cytometry, followed by unsupervised UMAP analysis (**Supplementary Figure 2**). We observed that the total CD3^-^CD19^-^CD56^+^HLA-DR^-^ NK cells were statistically similar in all groups (**Supplementary Figures 2A, B**; left violin plot). However, HLA-DR^+^CD56^+^ NK cells tended to be higher in Preg-INF or Preg-R compared with Preg-HC although not reaching significance (**Supplementary Figures 2A, B**; right violin plot). NK cell subsets based on CD3^-^CD19^-^CD56^+^HLA-DR^-^ antibody markers were then identified for their distribution and to highlight differences between groups using UMAP analysis (**Supplementary Figures 2C, D**). CD3^-^CD19^-^CD56^+^HLA-DR^−^ NK cells were then gated and categorized based on their CD56 and CD16 markers. We found that mature classical CD56^+^CD16^+^ NK II cells tended to decrease in Preg-INF or Preg-R compared to Preg-HC women (**Supplementary Figures 2D–F**; left violin plot); however, the difference was not significant (**Supplementary Figure 2F**). Furthermore, the number of non-NK cells tended to increase in Preg-INF and Preg-R (**Supplementary Figure 2F**; right violin plot). Overall, our data revealed that mature classical NK cells tended to be reduced, whilst the number of activated NK cells in Preg-INF and Preg-R compared with Preg-HC were increased.

### Subsided CD8^+^ T and NK cells in Preg-R were recapitulated in an independent geographical cohort

**Figure 3:**
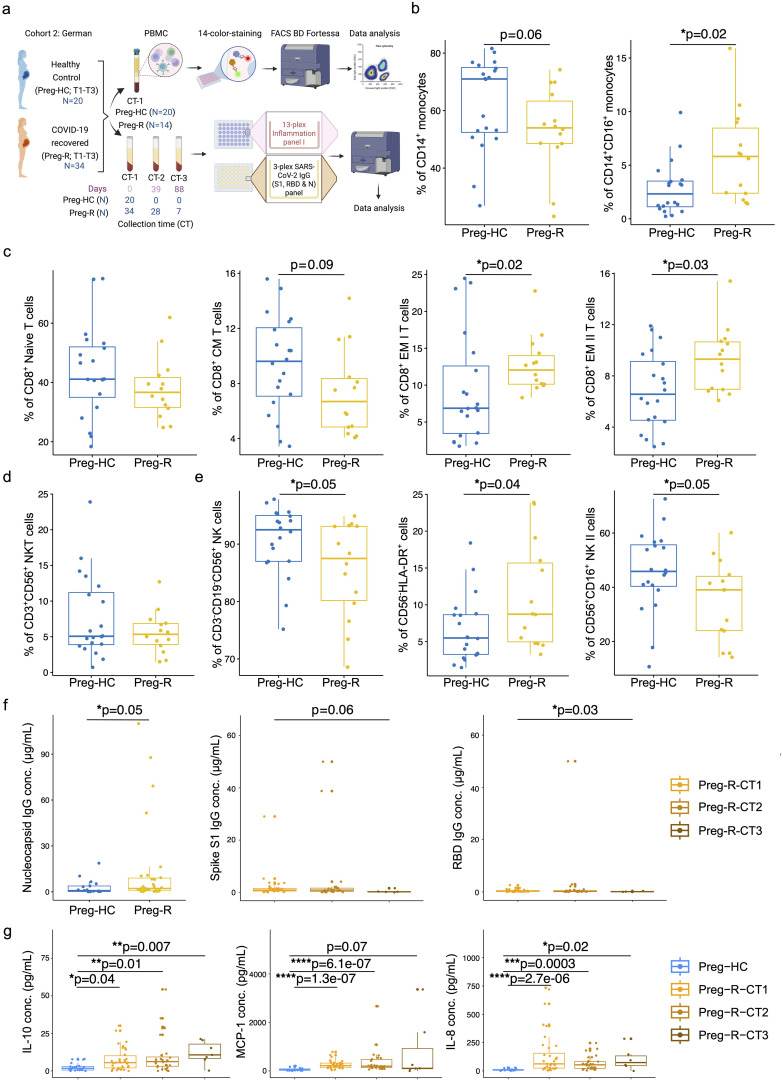
Validation of immunophenotyping studies and dysregulated humoral immune response in German cohort 2. **(a)** German Cohort 2: description of cohort and experimental plan using matched PBMCs and serum from pregnant healthy controls (Preg-HC) and COVID-19 recovered (Preg-R) pregnant women. **(b)** Reduced percentage of CD14^+^ monocytes and significantly increased CD14^+^CD16^+^ in Preg-R patients. Box plots show median, interquartile range (IQR), and the whiskers corresponding to the highest and lowest points within 1.5 times of IQR. Each dot represents an individual sample. Wilcoxon rank-sum test was used for p-value significance to compare pregnant healthy control (Preg-HC) and COVID-19 recovered (Preg-R). P ≤0.05 considered significant (*p ≤0.05). **(c)** Box and whisker plot representing the percentage of Naïve, CM, EM I, and EM II CD8^+^ T cells in Preg-HC and Preg-R patients. CD8^+^ Naïve and CD8^+^ CM T cells were lower in Preg-R. CD8^+^ EM I and CD8^+^ EM II T cells were significantly increased Preg-R. Wilcoxon rank-sum test was used for p-value significance. P ≤0.05 considered significant (*p ≤0.05). **(d)** Box and whisker plot representing NKT cells in Preg-R compared with Preg-HC. **(e)** Box and whisker’s plot showing reduced CD56^+^ NK cells and CD56^+^CD16^+^ NK II cells in Preg-R. Increased levels of CD56^-^HLA-DR^+^ lymphoid cells were significantly increased in Preg-R. Wilcoxon rank-sum test was used for p-value significance. P ≤0.05 considered significant (*p ≤0.05). **(f)** High levels of Nucleocapsid IgG antibody levels in serum of Preg-R group compared to Preg-HC (left graph). Wilcoxon rank-sum test was used for p-value significance. P ≤0.05 considered significant (*p ≤0.05). Spike S1 (middle graph) and RBD IgG concentration (ug/mL; right graph) at different collection time-points (CT) points (CT1, 0 day; CT2, 39 days; and CT3, 89 days post infection) during their pregnancy. Decreased levels of Spike S1 and RBD IgG antibodies 89 days post first collection time point in Preg-R group. Each dot represents an individual. Kruskal–Wallis test, adjusted for Dunn’s multiple comparisons test. P ≤0.05 considered significant (*p ≤0.05). **(g)** Examination of IL-10, MCP-1, and IL-8 levels in the serum of recovered women up to 89 days post infection. Each dot represents an individual on a box-whisker plot. CT1, 0 day; CT2, 39 days; and CT3, 89 days post infection-during their pregnancy Kruskal–Wallis test, adjusted for Dunn’s multiple comparisons test. P ≤0.05 considered significant (*p ≤0.05, **p ≤0.01, ***p ≤0.001, ****p ≤0.0001).

Immunophenotyping of SARS-CoV2-infected pregnant and recovered pregnant women from different countries has been performed and provided us with an unclear consensus on our understanding of immune cell type and changes due to the limited sample size and variable experimental designs (17, 32, 35, 43–45). Therefore, we validated our data obtained from Cohort 1 (Malaysian) with samples from Cohort 2 (German) collected from the University Hospital Tübingen, Germany, which was collected during the early pandemic in 2020–2021 (**Figure 3a**).

Based on our 14-color immunophenotyping, we identified that differences in monocytes, CD8^+^ T cells, and NK cells were restated in validation Cohort 2. Total CD14^+^ classical monocytes tended to be lower in recovered pregnant patients (Preg-R) than in healthy (Preg-HC) pregnant women (**Figure 3b**, left); however, intermediate monocytes (CD14^+^CD16^+^) were significantly more abundant in recovered pregnant women than in healthy pregnant controls (**Figure 3b**, right). We observed that naïve CD8^+^ T and CD8^+^ CM T cells tended to be lower, whereas levels of both CD8^+^ EMs (I and II) T cells in recovered pregnant women were significantly increased, reaching statistically significant levels (**Figure 3c**). No differences were observed in CD3^+^CD56^+^ NKT cells (**Figure 3d**). Furthermore, total NK and NK type II cells were significantly reduced (p = 0.05) in the Preg-R group compared with Preg-HC women (**Figure 3e**), while CD56^-^HLA-DR^+^ myeloid cells (p = 0.04) were significantly increased (**Figure 3e**). Therefore, our findings support the observation that NK cells occur at a lower frequency in recovered pregnant women.

### Dysregulated cytokines and chemokines are seen up to 89 days post infection

To gain a more granular view of the deregulated immune system, we selected a subset of inflammatory mediators for analysis using a multiplex-cytokine assay. To address this, we examined the serum of pregnant women after recovery at different collection time (CT) points (CT1: 0 day, CT2: 39 days, and CT3: 89 days post-infection recovery) during their pregnancy. Serological examination from the first time collection (CT1) point of serum suggested that viral nucleocapsid (N) IgG antibody levels were significantly upregulated in the Preg-R group compared to the Preg-HC group, as expected (**Figure 3f**; left plot; **Supplementary Figure 3A**). The nucleocapsid (N) IgG antibody data verified that the recovered group used in this study had an active SARS-CoV-2 infection before samples were collected. Additionally, the estimation of spike S1 IgG levels tended to be reduced without reaching significance, except for viral receptor binding domain (RBD) IgG levels in the Preg-R group (**Figure 3f**; middle and right plots).

We found that the anti-inflammatory cytokine IL-10 was consistently higher in recovered pregnant women at all collection time points than in healthy (pregnant) controls (**Figure 3g**). In contrast, proinflammatory chemokines such as MCP-1 and IL-8 levels were significantly higher up to 39 days from the first collection point, whereas only IL-8 levels were significantly higher at CT3 from the first collection point (**Figure 3g**). Furthermore, we identified that many other pro-inflammatory cytokines, including IFN-γ, IL-1β, TNF-α, IL-6, IL-12p70, and IL-23, were significantly upregulated in Preg-R women at least by one or two time points compared with the Preg-HC group (**Supplementary Figure 3B**). IFN-2α levels tended to be higher in the Preg-R group than in the Preg-HC goup, while the alarmin cytokine IL-33 was significantly upregulated in Preg-R at 89 days compared to Preg-HC women (**Supplementary Figure 3B**). Overall, cytokine and chemokine levels were persistently higher in recovered pregnant women up to 89 days post infection.

### Single cell gene expression profiling of Preg-R and Preg-HC

**Figure 4:**
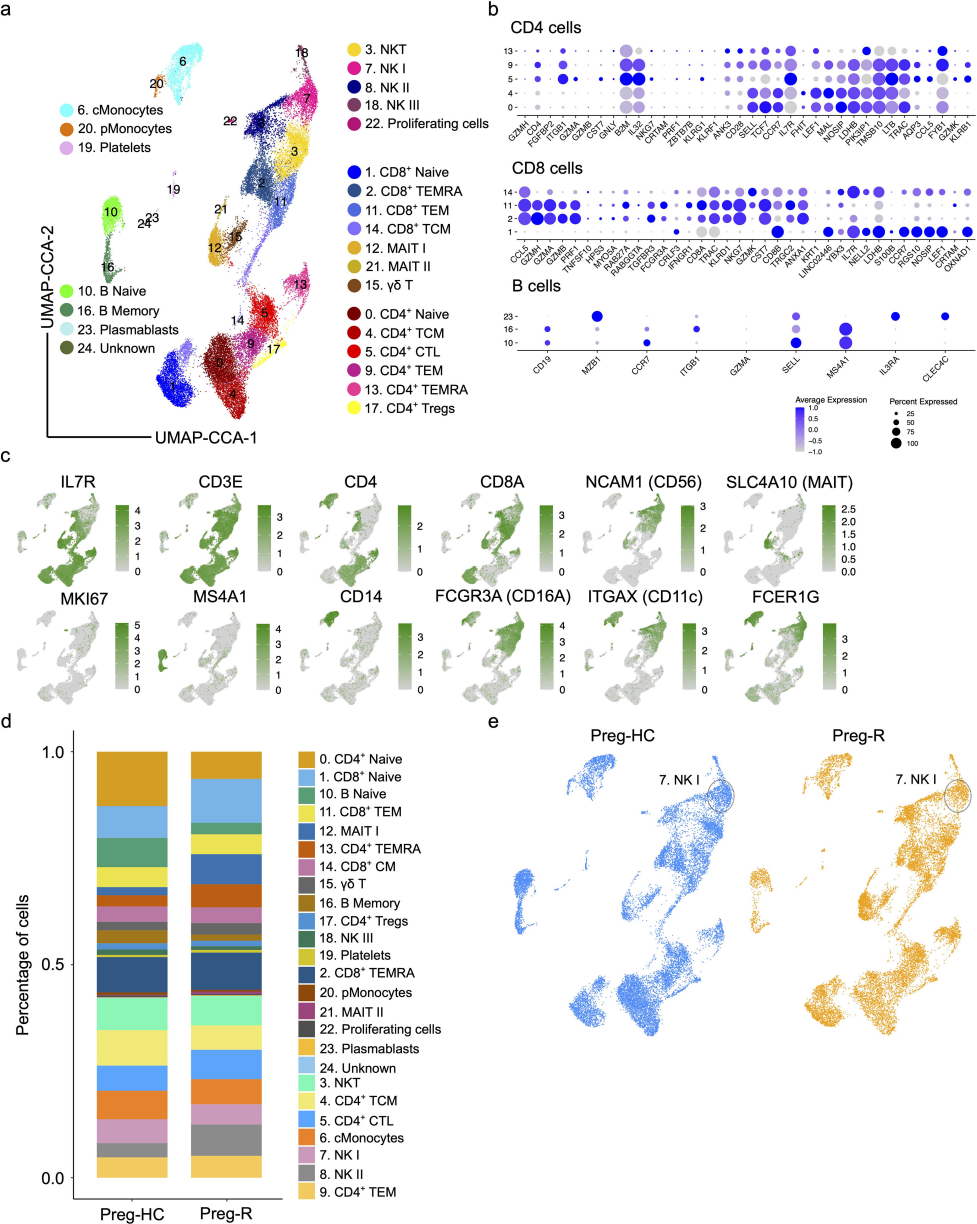
Single cell atlas and immune cell composition of healthy controls and recovered COVID-19 pregnant women. **(a)** Integrated UMAP (UMAP-CCA) of 30,394 cells derived from PBMCs. Sc-RNA-seq data were analyzed using Seurat pipeline. UMAP plots shown the different immune cell subsets based on distinct gene expression. Cell types are color-coded as shown in UMAP. **(b)** Dot plots show the expression level of canonical cell markers used to assign cell subset identification for major cell type including CD4^+^ T, CD8^+^ T, and B cells. **(c)** Feature plots show the key canonical markers used for identification of individual cell types. Green intensity shows increasing expression. **(d)** Percentage of major cell types in Preg-HC and Preg-R patients. **(e)** UMAP analysis of individual Preg-HC (blue) and Preg-R (orange) patient groups which highlight a reduced NK I cell population.

Flow cytometry enables us to gain insight at the protein level; however, due to the limitations of fluorochromes, it is not possible to explore the expression of other molecules that may be affected in Preg-R patients. Therefore, we employed single-cell scRNA-seq gene expression profiling using the 10× chromium Gel Bead-in-Emulsion method to investigate the heterogeneity of the immune cell landscape. We used four independent Preg-HC and two independent Preg-R samples in duplicate for scRNA-seq experiments. The UMAP plots show the data before- and after integration of all samples and batch correction (**Supplementary Figure 4**). We identified 25 subtypes of different immune cells (**Figures 4a–c**, **Supplementary Figure 5**). Furthermore, interrogation of cluster-specific gene expression for CD4^+^, CD8^+^, and B cells was performed according to their respective canonical marker genes (**Figures 4a–c**, **Supplementary Figure 5**). Individual gene markers for major cell subsets (CD4^+^ and CD8^+^ T cells, NK, MAIT, monocytes, DCs, and B cells) were demonstrated in the UMAP plots, which further confirmed the identification of each cell cluster (**Figures 4b, c**). Moreover, we identified a decreased percentage of the subsets classical Monocytes (cMonocytes), CD4^+^ naïve T cells, CD4^+^ CM T cells, naïve B cells, and memory B cells. However, the number of CD8^+^ naïve T cells, MAIT cells, and CD4^+^ TEMRA cells increased in Preg-R (**Figure 4d**). For NK cell subtypes, NK I cells were decreased, while NK II cell subtypes were increased in Preg-R compared with Preg-HC (**Figure 4d**). Moreover, discordance in the number of NK I cells was still apparent in the Preg-R group (**Figure 4e**). Thus, we were able to verify the different immune cell subsets by employing scRNA-seq, and the subsets were congruent with the flow cytometry data presented.

### Differential gene expression and pathway regulation in classical monocytes

**Figure 5:**
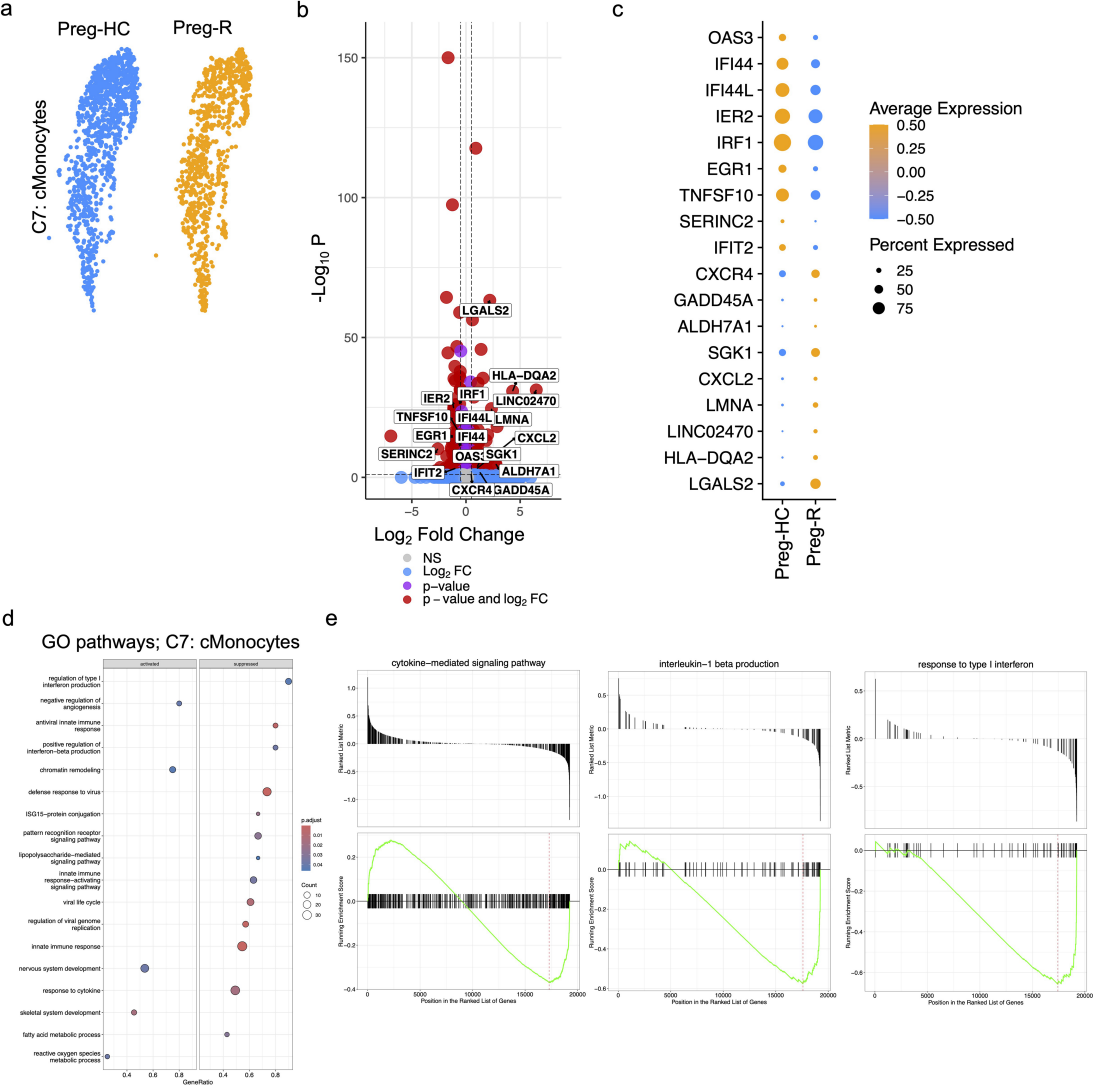
Activated classical (c)Monocytes in recovered pregnant patient group. **(a)** UMAP subplots for cMonocytes of Preg-HC and Preg-R patients. **(b)** Differentially expressed genes (DEGs) analysis in cMonocytes in Preg-R vs Preg-HC. Volcano plots showed the significantly upregulated and downregulated genes. **(c)** Dot plot shows the top 10 highly upregulated and downregulated genes in cMonocytes. **(d)** GSEA pathway analysis based on differentially expressed genes in cMonocytes obtained from Preg-R vs Preg-HC based on a bubble plot (left; activated and right; supressed). **(e)** GSEA plots show selected pathways (cytokine-mediated signaling pathway, interleukin-1 beta production, and response to type I interferon).

Classical monocytes (cMonocytes) are the principal innate immune cells required for defence as well as being the main culprit participating in the ‘cytokine storm’ during active SARS-CoV-2 infection (42, 46, 47). We observed a clear and reduced number of cMonocytes in Preg-R compared to Preg-HC (**Figure 5a**). To provide further insight into the genes that drive this change, we explored the differentially expressed genes (DEGs) of cMonocytes in Preg-R compared with Preg-HC. Our data revealed that 161 genes were upregulated, while 289 genes were downregulated (fold change ≥0.20, FDR value ≤0.05) in Preg-R women (**Figure 5b**). Among the most prominently upregulated genes were *OAS3, IFI44, IFI44L, IER2*, *EGR1*, *TNFSF10, SERINC2*, and *IFIT2*, while the key downregulated genes were *CXCR4, GADD45A, ALDH7A1, SGK1, CXCL2, LMNA, LINC02470, HLA-DQA2*, *and LGALS2* (**Figures 5b, c**; volcano plot and dot plot). GSEA data revealed that these attenuated networks were significantly enriched in genes involved in the regulation of type I interferon production, antiviral innate immune response, ISG-15-protein conjugation, positive regulation of interferon-beta production, defense response to virus, viral life cycle, regulation of viral genome replication, pattern recognition receptor signaling pathways, response to cytokines, and fatty acid metabolic processes (**Figure 5d**). Negative regulation of angiogenesis, chromatin remodeling, nervous system development, and skeletal system development was upregulated in Preg-R women (**Figure 5d**). The GSEA score data suggested that the cytokine-mediated signaling pathway, interleukin-1 beta production, and response to type 1 interferon were decreased in Preg-R compared to Preg-HC (**Figure 5e**). Overall, our data revealed that cMonocytes were less abundant in Preg-R. Critically, we observed that post-SARS-CoV-2 infection, classical monocytes are deregulated in Preg-R patients, pointing to the wholesale reprogramming of several signaling pathways leading to a compromised immune function in the convalescence period.

### Dysregulated gene expression of B cells, CD4^+^ T cells and MAIT cells in Preg-R patients

**Figure 6:**
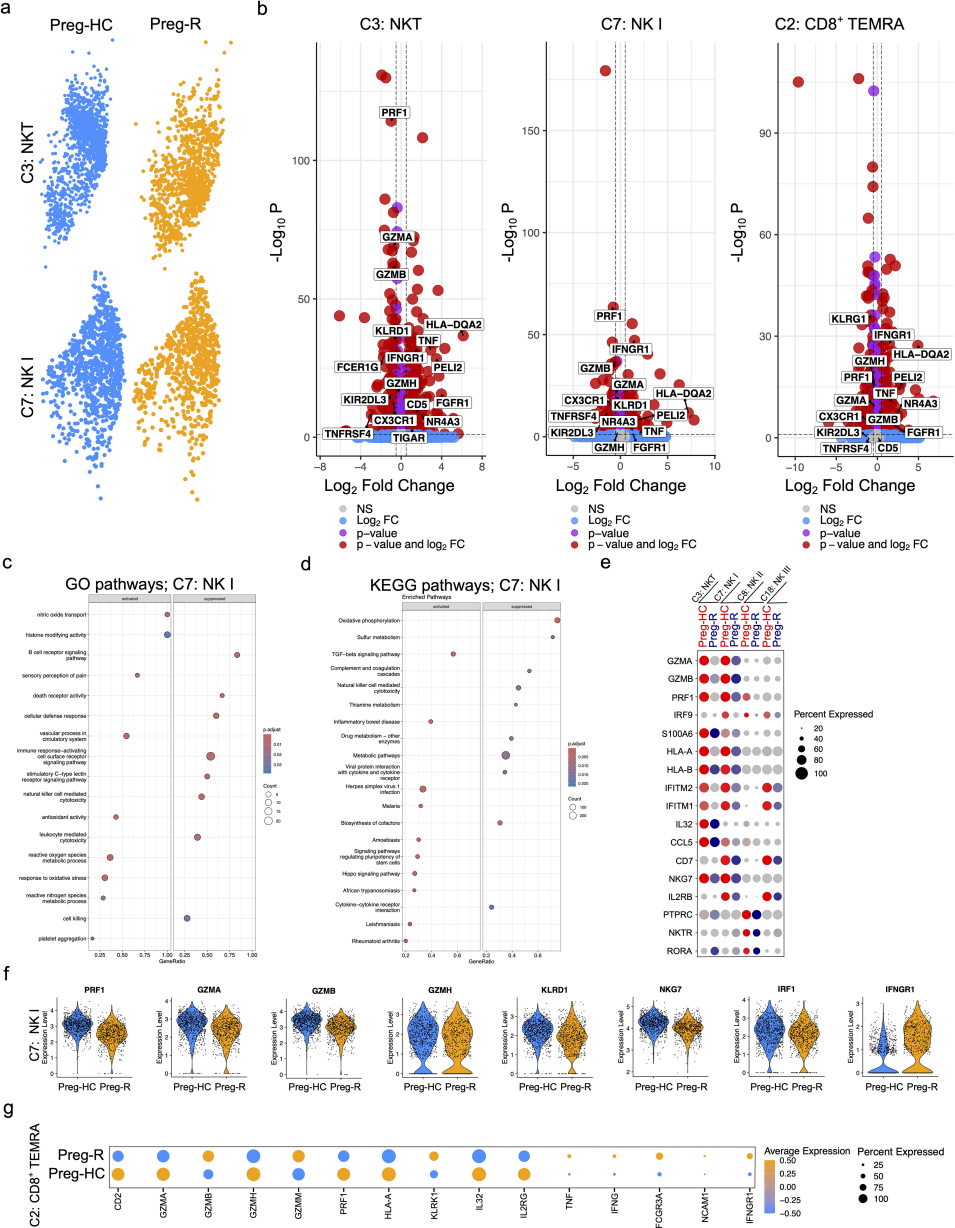
Reduced cytotoxic functions of NKT and NK cells in recovered pregnant women. **(a)** UMAP plots show the NKT (upper panel) and NK I (lower panel) cells from pregnant healthy controls and recovered patients. **(b)** Differential gene expression analysis of NKT, NK I, and CD8^+^ TEMRA cells using volcano plots. **(c)** GSEA pathway enrichment analysis for NK (I) Selected activated and suppressed pathways are shown on GSEA bubble plots. **(d)** KEGG pathway analysis of NK I cells. Most significantly pathways are shown on the GSEA plot. **(e)** Dot plot represents the selected cytotoxic function expressing genes in NKT (C3), NK I (C7), NK II (C8), and NK III (C18) cells. **(f)** Violin plots show significantly regulated genes related with cytotoxic functions in NK I cells. Each represents one cell. **(g)** Dot plot representing genes related with cytotoxic functions in CD8^+^ TEMRA (C2) cells.

Both naïve and memory B cells, which are involved in the humoral immune response, were likewise reduced in Preg-R (**Supplementary Figure 6**). DEG analysis highlighted that several genes were subtly dysregulated (55 upregulated and 89 downregulated) in B-naïve cells, and 66 upregulated and 148 downregulated genes in B memory cells (**Supplementary Figures 6A, B**). GSEA revealed that CXCR chemokine receptor binding and IL-8 production were reduced in B-naïve cells in Preg-R compared with control samples (**Supplementary Figures 6C**; left GSEA plot, **Supplementray Figure 6D**; right plot). Similarly, the GSEA pathway analysis in B memory cells revealed that type I interferon-mediated signaling, regulation of IL-4 production, IL-8 production, and IgA immunoglobulin complex pathways were reduced in Preg-R compared to Preg-HC, while the natural killer cell activation pathway, transmembrane receptor activity, and cellular defense response were upregulated in Preg-R (**Supplementary Figures 6C, E**; right GSEA plot).

CD4^+^ T helper cells support B cells, CD8^+^ T cells, and other immune cells (48). In the Preg-R group, CD4^+^ T cells diverged into two subpopulations, represented by a reduction in naïve CD4^+^ T cells and an increase in CD4^+^ T cytotoxic lymphocytes (CD4^+^ CTLs) (**Supplementary Figure 7**). Thus, we explored the gene signature of naïve CD4^+^ T cells and CD4^+^ CTLs and identified 316 upregulated genes, 231 downregulated genes in naïve CD4^+^ T cells, and 308 upregulated genes, and 323 downregulated genes in CD4^+^ CTLs respectively (**Supplementary Figures 7A, B**). The top 15 upregulated (*PELI2, FOSL2, NK7, HIST1H1E, SLC12A6*, and *GPRIN3*) and downregulated (*IRF1, FCGRT, CST3, GNAI2, IL2RG*, and CARD16) genes are shown as dot plots for all CD4^+^ T cell subsets, including CD4^+^ naïve T cells (C0) cells, CD4^+^ CM T cells (C4), CD4^+^ CTLs (C5), CD4^+^ EM T cells (C9), CD4^+^ TEMRA cells (C13), and CD4^+^ CM Tregs (C17) (**Supplementary Figure 7C**). The key cytokines controlling transcription factors IRF9 and IRF1 were downregulated, while *RORA* was upregulated in the Preg-R group compared to the control group Preg-HC in CD4^+^ CTLs (**Supplementary Figure 7C**; violin plots). GSEA analysis of CD4^+^ naïve T cells revealed that the mitochondrial respiratory chain complex I and NADH dehydrogenase complex pathways were suppressed while killing the cells of another organism, and cell killing pathways were activated (**Supplementary Figure 7D**; left). In CD4^+^ CTLs, proton-motive force-driven mitochondrial ATP synthesis and ATP biosynthetic processes were suppressed, whereas the transcription regulatory region nucleic acid binding and negative regulation of RNA metabolic process pathways were activated (**Supplementary Figure 7D**, right). However, a closer inspection of the GSEA pathway data revealed that natural killer cell activation pathway was activated in CD4^+^naïve T cells (**Supplementary Figure 7E**; left). In CD4^+^ CTLs, histone H3 methyltransferase activity, T cell differentiation involved in the immune response, and T-helper 17 type immune response pathway genes were upregulated (**Supplementary Figure 7E**; right GSEA plot).

A previous study suggested that dysregulated MAIT cells contribute to COVID-19 in pregnant and nonpregnant women (32). MAIT cells express receptors for type I IFNs, IL-12, IL-15, and IL-18; thus, these cells could potentially be activated by proinflammatory cytokines (49). MAIT cells, which are involved in the direct recognition of peptides without MHC molecules, were increased (% cell fraction) in Preg-R compared with Preg-HC (**Figure 4d**, **Supplementary Figure 8A**; left UMAP plot). Thus, we investigated gene expression in MAIT cells and identified that several genes were differentially expressed in Preg-R (250 upregulated and 162 downregulated genes) compared to Preg-HC (**Supplementary Figure 8A**; right volcano plot). GSEA pathway analysis suggested that numerous pathways were upregulated in Preg-R compared with Preg-HC, which are related to nitric oxide, IgM immunoglobulin complex, and hydrogen peroxide catabolic processes (**Supplementary Figures 8B, C**). Thus, MAIT cells are likely to be involved in the host immune response against SARS-CoV-2 infection in pregnant women.

### NKT, NK, and CD8^+^ TEMRA cells had reduced cytotoxic function related genes and pathways

Previously, we and others have reported that NK cells and CD8^+^ T cells were decreased in moderately diseased and recovered COVID-19 patients, albeit in non-pregnant patients, and that the decrease in these particular cell types was correlated with increased disease severity (40, 50, 51). Therefore, we examined the gene markers related to NKT (C3), NK I cells (C7), and CD8^+^ TEMRA (C2) function in pregnant women who had recovered after SARS-CoV-2 viral infection. Consistent with previous observations, our DEG analysis of NKT cells revealed that 483 genes were upregulated, and 609 genes were downregulated in the Preg-R group compared with Preg-HC (**Figures 6a, b**; left panel). Furthermore, NK I cell types had 269 genes upregulated and 330 genes repressed, similarly, CD8^+^ CLTs followed a similar pattern, with 413 genes upregulated and 608 genes downregulated (**Figures 6a, b**; middle and right panels). GSEA from NK I cells suggested that several pathways such as the B cell receptor signaling pathway, death receptor activity, cellular defense response, immune response-activating cell surface receptor signaling pathway, stimulatory C-type lectin receptor signaling pathway, natural killer cell-mediated cytotoxicity, and leukocyte-mediated cytotoxicity were suppressed, while nitric oxide transport, histone modifying activity, sensory perception of pain, vascular processes in the circulatory system, antioxidant activity, reactive oxygen species metabolic process, response to oxidative stress, and platelet aggregation were activated in Preg-R compared to Preg-HC (**Figure 6c**). Furthermore, KEGG pathway analysis identified that oxidative phosphorylation, natural killer cell-mediated cytotoxicity, complement and coagulation cascades, metabolic pathways, and viral protein interaction with cytokine and cytokine receptors were suppressed, while TGF-beta signaling was activated in NK I cells (**Figure 6d**). We examined the genes related to cytokines, chemokines, and cytotoxic functions in NKT (C3), NK I (C7), NK II (C8), and NK III (C18) cells, and presented in a dot plot (**Figure 6e**). Most of the NK I cytotoxic function-related genes (*PRF1, GZMA, GZMB, GZMH, KLRD1, NKG7*, and *IRF1*) were significantly downregulated, while IFNGR1 was significantly upregulated in Preg-R compared with Preg-HC (**Figure 6f**). Taken together, our data reveal that NK I cells have a reduced cytotoxic function gene signature and deranged interferon signaling implicating the deleterious function of these cell types post-SARS-CoV-2 infection. Moreover, in the case of CD8^+^ TEMRA (C2) cells, several cytokine signaling pathway-related genes were upregulated, whereas the cytotoxic function pathway genes were downregulated. The most common genes related to cytotoxic functions included *CD2, GZMA, GZMH, PRF1, HLA-A, IL-32*, and *IL2RG*, which were significantly downregulated, and *GZMB, GZMM, KLRK1, TNF, IFNG, FCGR3A, ICAM1*, and *IFNGR1* that were significantly upregulated in Preg-R compared with Preg-HC (**Figure 6g**).

### Attenuated antiviral activity of major immune cell subsets in recovered pregnant women

**Figure 7:**
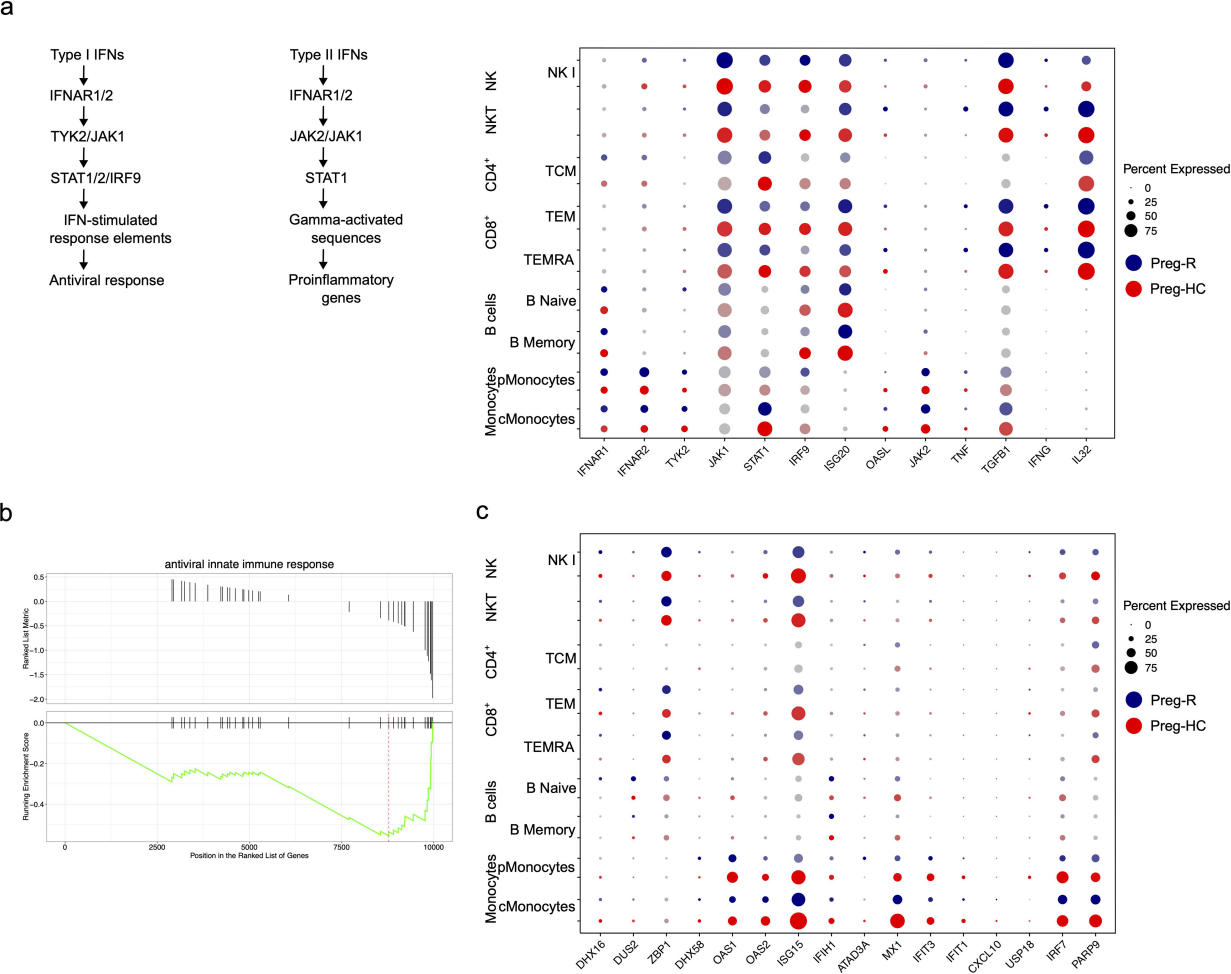
Dysregulated Type I and type II IFNs signalling in recovered pregnant women. **(a)** Schema of Type I and II signaling pathways. Dot plot showing Type I IFNs signalling genes (*IFNAR1, IFNAR2, TYK2, JAK1, STAT1, IRF-9, ISG20*, and *OASL*) and their corresponding expression level. Type II IFNs signaling genes (*IFNG, IL32, JAK2, TNF*, and *TGFBI*). **(b)** GSEA plot for antiviral innate immune response. **(c)** Antiviral innate immune response and expressed genes in different immune cell subsets using dotplot.

In the general population (non-pregnant), post-SARS-CoV-2 infection immunity decreases (52, 53). Thus, we explored the status of the immune response in pregnant women who recovered from viral infections using different immune cell subsets. To protect the host from infection, cardinal genes involved in virus defense, such as *ISG20, OASL, OAS3, TNF, IRF9*, and *STAT1*, were manually mined *in silico*. We discovered that the expression of the type I interferons (IFNs) receptor *IFNAR1* was decreased in numerous major immune cell subsets (CD8, and NK cells); however, *IFNAR2* appeared to be drastically repressed in the B cell population (**Figure 7a**). Downstream of the type I IFNs signaling pathway, *TYK2, JAK1, STAT1, IRF9, ISG20, ISG20L2*, and *OAS3* levels were also decreased in pMonocytes (**Figure 7a**). Moreover, type II IFNs signaling molecules, such as IFN-γ, were increased in cytotoxic T cells and NKT cells. TNF-α levels were reduced in monocytes, whereas cytotoxic CD8^+^ T cells and NKT cells showed increased levels (**Figure 7a**). We further observed that *TGFB1* expression was upregulated in cMonocytes, CD8^+^ T and NK cells (**Figure 7a**). Next, we explored the role of antiviral activity of innate and adaptive immune cell subsets in recovered pregnant women. We identified that antiviral activity pathway genes (*OAS1, OAS2, ISG15, MX1, IFIT3, IRF7*, and *PARP9*) were downregulated in Preg-R women in most immune cells, including monocytes, CD8^+^, NK, and NKT cells, respectively (**Figures 7b, c**). Overall, the data suggest a decrease in the pro-inflammatory signaling mechanism and upregulation of anti-inflammatory molecules in Preg-R women.

### Validation of reduced cytotoxic functions and cytokine receptor expression in NK cells

**Figure 8:**
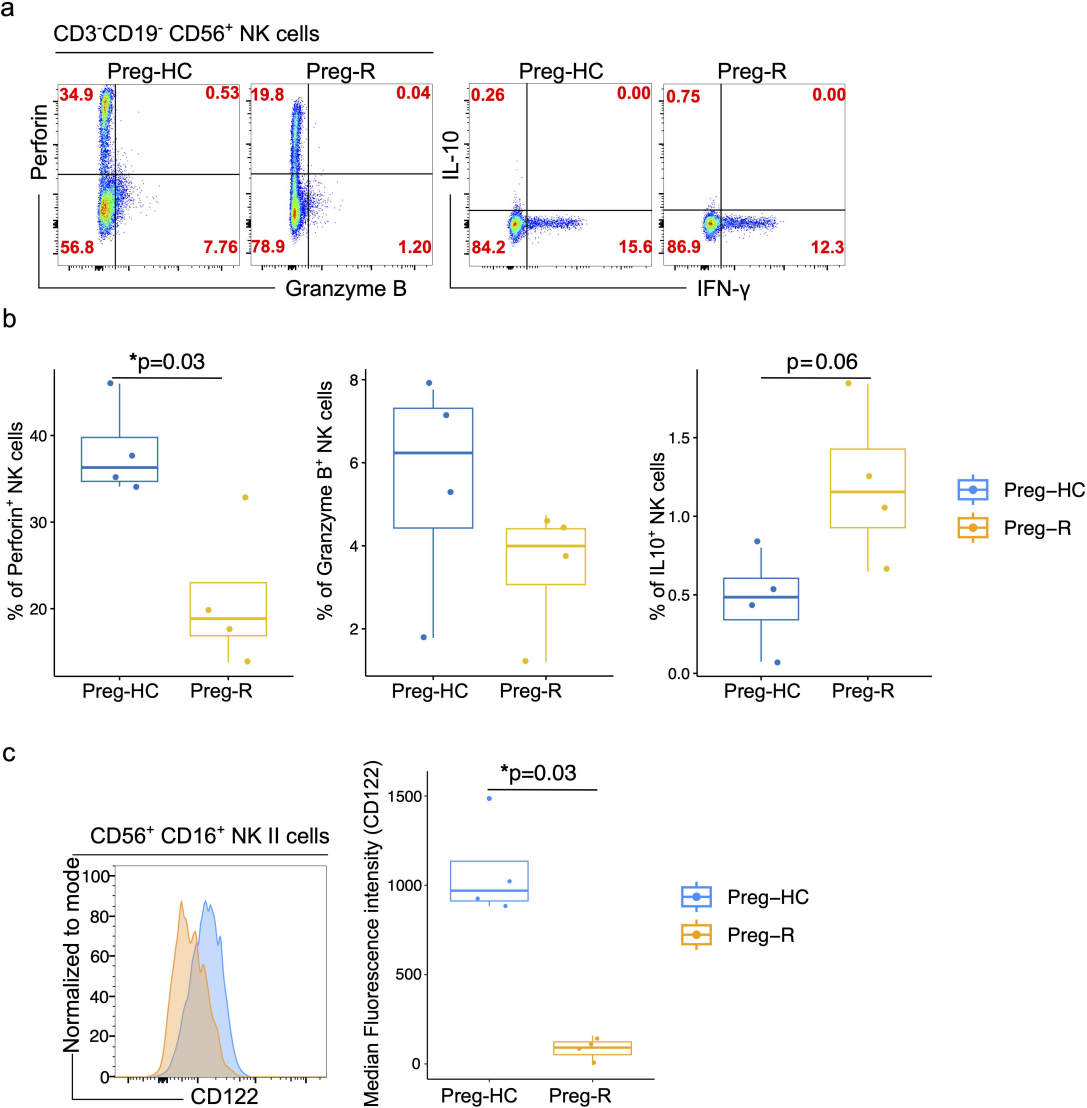
Validation of NK cell functions molecules by flowcytometry. **(a)** FACS plots represents the expression of perforin, granzyme B, IL 10 and IFN-γ in CD3^-^CD19^-^CD56^+^NK cells in Preg-HC and Preg-R groups. **(b)** Box plots represent the statistical significance of perforin, granzyme B, IL 10 levels in NK cells. **(c)** FACS histogram plot presents the expression of CD122 on CD56^+^CD16^+^ classical NK cells. Preg-HC and Preg-R samples overlayed on each other for identification of CD122 expression for comparative purpose and expression was normalized to mode (left FACS plot). Box and Whisker plot represents the median expression of CD112 marker. Wilcoxon rank-sum test was used for p value significance. P ≤0.05 considered significant (*p ≤0.05).

We identified reduced cytotoxic gene expression in NK cells; thus, we explored which molecules are involved in the functionality of these cells. We performed flow cytometric analysis of perforin, granzyme B, IL-10, and IFN-γ by intracellular staining. We observed that perforin expression in NK cells was significantly lower in the Preg-R group than in the Preg-HC (**Figures 8a, b**). Granzyme B levels were reduced, and we observed increased levels of IL-10 (**Figures 8a, b**), although both did not reach statistical significance. No changes in IFN-γ levels were observed.

Pregnancy represents a transient state of immunosuppression that maintains a balance between maternal immunity and the fetus. Furthermore, pregnant women are at higher risk of (viral) infection or reactivation of common opportunistic viruses such as cytomegalovirus (CMV) owing to physiological immunosuppression (3–7). Compelling evidence suggests that CMV infection impairs NK cell function *via* loss of monocytes, albeit in a murine model (54, 55). In brief, the response to CMV infection occurs in waves. In response to infection, plasmacytoid dendritic cells (pDCs) produce Type I interferons (Type I IFNs), constituting the first line of defense. The critical second (and third) waves of the immune response block viral replication by killing infected cells *via* NK cells (55). Inflammatory mediators including IL‐12 and IL‐15 which are secreted by conventional dendritic cells (cDCs), can indirectly bolster viral defense by stimulating NK cell proliferation, activation, and effector functions (55, 56). We posited that the reduced NK cytotoxic function due to SARS-CoV-2 infection could follow a similar pattern. Therefore, we explored the expression of IL-15RA (CD215) and IL-15RB (CD122) in monocytes and NK cells, respectively, to estimate the dynamics of NK cell function in post-COVID 19 pregnant women. The expression of IL-15RA (CD215) on CD14^+^ monocytes was reduced in Preg-R compared with that in Preg-HC; however, the difference was not statistically significant (**Supplementary Figure 9**). Furthermore, the levels of IL-15RB (CD122) in classical NK cells were significantly reduced in Preg-R compared to Preg-HC (**Figure 8c**). In summary, our data provides evidence that NK cells in recovered COVID-19 Preg-R women have reduced cytotoxic function and cytokine receptor expression, which are involved in their effective function.

## Discussion

Pregnancy is a complex physiological phenomenon, and the host immune system must sustain a balance to enable pregnancy to ensue and protect the mother from infections (1, 2). Data from other infectious outbreaks (Influenza, Zika, or Ebola virus) have highlighted that pregnant women are more susceptible, develop more severe complications, and have adverse pregnancy outcome rates (7). Severe maternal COVID-19 infection is associated with an elevated risk of poor neonatal Apgar scores and maternal mortality (22–24). However, the mechanisms underlying this increase in susceptibility during pregnancy are not well understood. Furthermore, conflicting findings in earlier studies due to limitations such as small cohort sizes, timing of sample collection/tubes, heterogeneity in defining appropriate controls (pregnant and non-pregnant), new SARS-CoV-2 clades, maternal ethnicities, co-morbidities, and social determinants (poverty, overcrowding, or air pollution) compromise our full understanding of the modulatory effects of the immune response to SARS-CoV-2 during pregnancy.

To gain deeper insights into how in pregnancy immune system is affected during and following SARS-CoV-2-viral infection, we characterized the response using PBMCs and serum collected from gestationally age-matched pregnant women during the acute and convalescent phases of SARS-CoV-2 infection compared to pregnant SARS-CoV-2-negative controls. In this report, using multi-color flow cytometry and scRNA-seq based on two different geographical (one high-income and one low-middle-income country setting) cohorts, we identified a highly detailed immune fingerprint during SARS-CoV-2 infection and in post-COVID-19 pregnant women. Our multi-color immunophenotyping data reveals that there was a significant reduction in the percentage of total lymphocytes, whilst with there was a pronounced decrease in monocytes in SARS-CoV-2 infected and recovered pregnant women compared with healthy pregnant women. Our findings are further corroborated by recent data suggesting that in recovered pregnant women, there was indeed a reduction in lymphoid cells compared with healthy pregnant women (32). Furthermore, our characterization of CD4^+^ T cells and CD8^+^ T cells revealed an increased percentage of early or late effector CD8^+^ T cells in pregnant SARS-CoV-2 infected women compared with post-COVID-19 pregnant women, consistent with a recent publication (35). Moreover, a reduced trend of effector cells, either CD4^+^ or CD8^+^ T cells, was also observed when comparisons were made between healthy pregnant controls and SARS-CoV-2 infected pregnant women. Taken together, these results indicate that a viral infection follows a J-shaped curve, leading to a transient reduction of effector cells, which are then increased once the virus is cleared (57). Our data agree with other published findings that indicate that those with moderate infection had an increased reappearance of effector T cells compared with patients with severe COVID-19 (58).

Earlier studies on NK cells did not reach a consensus (31, 45). Our phenotyping data suggested that there was a decreased fraction of NK cells. Furthermore, functional analysis also revealed that NK cells in recovered pregnant women have reduced cytotoxic proteins and lower turnover of respective mRNA levels using flow cytometry and scRNA-seq, respectively, which were similar in both cohorts. We postulated that this reduction in NK cells is required to prevent an exacerbated immune response during active infection of pregnant SARS-CoV-2 women. Cytokines and chemokines detected in the plasma of pregnant SARS-CoV-2 infected women have revealed reduced levels of IL-12(p70) and increased RANTES levels (31). Based on these results, the authors speculated that suppression of cytokine and chemokine levels could be a strategy to avoid adverse pregnancy outcomes and may represent an anti-SARS-CoV-2 response during pregnancy. In contrast, our findings suggested that both pro-inflammatory cytokines and chemokines (IFN-γ, IL-1β, TNF-α, IL-6, IL-12p70, IL-23, IL-33, MCP-1 and IL-8) and anti-inflammatory cytokines (IL-10) were upregulated in pregnant recovered women. Therefore, it appears that even up to 90 days post COVID-19 infection, increased inflammation persists in otherwise recovered pregnant women, the long-term effect of this ‘out of phase’ inflammation remains unknown. Maternal co-infections with human immunodeficiency virus (HIV) reshape the *in utero* environment through changes in secretion or inhibition of inflammatory mediators (59). Pregnant women with HIV have high levels of inflammation and residual immune dysfunction (decrease in CD4^+^ T cells); even with the use of effective antiretroviral therapies (ARTs), the culmination of both disrupts fetal immune homeostasis and alters long-term immune cell function in these children (60). Furthermore, these children are reported to have a higher risk of severe infections and infection-related hospitalization than HIV-unexposed children (59). The effect of maternal SARS CoV-2 infection on child health is under debate and is actively being researched.

To further investigate the heterogeneity of the immune cell landscape, we performed single-cell transcriptomics analysis. Our scRNA-seq data revealed that, in classical monocytes, most pathways related to cytokine-mediated signaling, IL-1β production, and response to type I IFNs were downregulated in post-COVID-19 pregnant women. Sureshchandra et al. also reported selective loss of type I IFNs signaling from decidual macrophages (35). These observations indicate that even mild infections can rewire the immune interface within the decidua, which has the potential for long-term adverse outcomes in neonates.

Regulation of host chromatin architecture is utilized by a variety of viruses to paralyze host defenses or to impose long-term influences, e.g., viral latency (61). In SARS-CoV-2 infection, the epigenome is also altered, including a global reduction in the active chromatin mark H3K27ac and a specific increase in H3K4me3 at proinflammatory gene promoters (62). Interestingly, these changes were unique to SARS-CoV-2 when compared to infection by common cold coronavirus or immune stimuli that did not elicit these changes (63, 64). Our data revealed that chromatin remodeling pathways were upregulated in post-COVID-19 pregnant women. Therefore, it is plausible that proinflammatory pathways are modulated by additional gene regulatory mechanisms in post-COVID-19 pregnant women.

NK cells communicate with B cells, and this crosstalk is required for their effective functioning during viral infection (65, 66). Our study suggests that memory B cells from recovered pregnant women have decreased expression of type I IFN-mediated signaling. Furthermore, the regulation of IL-4 and IL-8 production pathways was diminished, whereas the natural killer cell activation pathway was activated in B cells. Although we observed that NK cells were compromised in their function, the NK cell activation pathway was upregulated in B memory cells. Thus, SARS-CoV-2 infection could result in misdirected communication between NK and B cells, thereby contributing to altered immunocompetence (67). Therefore, an understanding of this complex interactive mechanism is required.

Immune cells are dependent on circulating nutrients in the blood, and changes in cellular metabolism affects the differentiation and effective function of T cell subsets, which can contribute to disease (68, 69). Briefly, following proinflammatory stimuli, immune cells increase glucose consumption. This leads to the accumulation of inflammatory metabolites that augment antimicrobial activity (70, 71). In the case of SARS-Cov2 infection in (non-pregnant) adults, we reported that in infection, intracellular metabolites from the glycolysis and oxidative phosphorylation (TCA cycle) pathways had a sustained reduction, which may represent an alternative strategy to meet the high demands of energy consumption to combat the ongoing viral infection (40). Our scRNA-seq data revealed that CD4^+^ T either naïve CD4^+^ T or CD4^+^ CTLs in recovered pregnant women suppressed mitochondrial respiratory chain complex I, NADH dehydrogenase complex, and ATP biosynthesis, respectively. A recent study provided compelling evidence that a tolerance defense strategy during serious infections exists by limiting active immune responses, partly by lowering glycolysis to avoid harmful hyperinflammatory responses (72, 73). Critically, the clinical practice of providing a continuous glucose supply leads to hyperglycemia, which might result in excessive immune cell glycolysis and disrupted pro- and anti-inflammatory balance (74). Correspondingly, individuals with diabetes or dysglycemia have higher rates of mortality and hospitalization (75). Moreover, we observed several upregulated pathways, including cytokine signaling (*TNF* and *IFNGR1*), histone H3 methyltransferase activity, post-transcriptional gene silencing, T cell differentiation involved in the immune response, and T helper cell 17 pathways in CD4^+^ CTLs gene signatures from recovered pregnant women. Intriguingly, alterations in cell metabolism are known to have profound effects on the epigenetic landscape, as well as the production of essential substrates for epigenetic modifications, thereby increasing the risk of (re)infections (76). Taken together, it appears that effector CD4^+^ T cells from recovered pregnant women have a competitive advantage in avoiding overt inflammation to protect against ensuing pregnancy. How the myriads of hormone-dependent signals and effectors are integrated to produce cell-specific and time-sensitive transcriptional responses remains a major mystery in pregnancy-related immune responses.

MAIT cells are referred to as antimicrobial T cells, operating as innate-like sensors and initiating antiviral responses; critically, they possess strong tissue-homing properties (77). In non-pregnant individuals, MAIT cells are reduced in SARS-Cov2 infection (45, 78). It has been reported that MAIT cells decrease in circulation but return to normal levels during convalescence. In our data, an increasing trend of MAIT cells was observed in convalescent pregnant women, and pathway analysis revealed that MAIT cells had increased reactive oxygen species and metabolic processes, while the immune response-regulating signaling pathway and IgD/E immunoglobulin complexes were suppressed. We also observed that MAIT cells had higher levels of CCR6 and CCL4 chemokines, which are required for tissue chemotaxis (79). We speculated that the higher chemotaxis signature identified in MAIT cells may be due to homing to the site of infection for pathogen clearance.

As mentioned above, innate immune cells, including NK cells, are also important for the clearance of infections by migrating to affected sites. In the general population, NK cells decrease in moderate and severe cases of SARSS-CoV-2 infection (80). Accordingly, we observed reduced NK cell abundance and a dysregulated gene expression profile in both cohorts. The most repressed gene ontology pathways were immune response-activating signaling, B cell receptor signaling pathways, death receptor activity, cellular defense response, and stimulatory C-type lectin receptor signaling pathways, whereas nitric oxide transport, histone modifying activity, response to oxidative response and platelet aggregation were induced. Interestingly, based on selective and relevant KEGG analysis, we identified that oxidative phosphorylation, natural killer cell-mediated cytotoxicity, viral protein interaction with cytokines and cytokine receptors, and metabolic pathways, were suppressed except for TGFB signaling and herpes simplex virus 1 infection, which were activated in pregnant recovered women. An in-depth analysis of individual gene markers related to cytotoxic function, such as *PRF1, GZMA, GZMB, GZMH, KLRD1, NKG7*, and *IRF1*, were suppressed in pregnant recovered women. To verify this dysregulation at the protein level, flow cytometry was performed. We found that NK cells had reduced perforin, granzyme B, and CD122 levels and increased intracellular IL-10 cytokine secretion. In parallel, serum IL-10 levels were upregulated in the recovered pregnant women. We posit that NK cells attempt to maintain inflammatory balance as a feedback mechanism. In agreement with this hypothesis, NK cells are known to dampen immune responses to several pathogens by producing IL-10 during systemic infection in a toxoplasmosis model (81). The dampened immune response may be beneficial to the host by protecting it from uncontrolled immune-mediated pathology of tissues and organs, or it can be detrimental to the host through the promotion of immunosuppression, and consequently, pathogen persistence and spread (82). IL-10-producing NK cells have been reported in chronic hepatitis C virus (HCV) infection, viremic HIV infection, chronic hepatitis B virus (HBV) infection, and sepsis (82). Furthermore, Clark et al., albeit in mice, found that IL-10 produced by NK cells increases susceptibility to systemic bacterial infection owing to a reduced number of monocytes. Another immunocompromised population includes those receiving hematopoietic cell transplantation (83). Kandalla et al. elegantly identified a coordinated differentiation program between myeloid and NK cells that plays a major role in reestablishing protection against viral infections and designated a crucial role of M‐CSF‐therapy in enhancing antiviral immunity (55). Currently, the therapeutic options for SARS-CoV2 infection in non-pregnant individuals are limited (Foscavir, Ganciclovir, Remdesivir, Cymeven), and their use in pregnancy is contraindicated as they may result in embryo/fetal teratogenesis or intrauterine death (84). We observed that the NK cell fraction was low and could result in vulnerability to infections in pregnant women, which could further impact their neonates. Therefore, the use of macrophage-colony stimulating factor (M-CSF)-driven improvement of NK cell functions (55) *via* IL-15 and Type I IFNs induction in monocytes could be applied as cytokine therapy in pregnant women and should be investigated in future scientific endeavors.

There are many parallels that can be drawn between our study and findings with other viral infections, including CMV, herpes, and HIV, all of which have profound effects on the maternal–fetal dyad. This vulnerability increases the risk of spontaneous abortion, premature birth, stillbirth, and/or maternal death (85). An earlier report highlighted that in mild/asymptomatic mothers, SARS-CoV-2 altered the transcriptional and functional state of the placenta and circulating fetal immune cells, thereby leading to adverse pregnancy outcomes (64). Meta-analyses performed by others have now provided strong evidence of *in utero* SARS-CoV-2 transmission in 3% of pregnancies (86) and advocate a strong correlation between disease severity with SARS-CoV-2 positivity in the fetus as well as poor obstetrical outcomes (miscarriage, preterm birth, stillbirths, and neonatal deaths) (86). Earlier in the pandemic, a case study identified that persistent placental infection (placenta was positive for nucleocapsid) was observed in an asymptomatic woman at eight weeks of gestation, leading to fetal demise (87). Thus, SARS-CoV-2 could lay dormant in non-respiratory tissues (e.g., the endometrium) through ‘stochastic seeding’ (88) and may contribute to the post-acute sequelae of SARS-CoV-2. Furthermore, since SARS-CoV-2 may suppress the immune system, it may be sufficient to reactivate dormant infections such as Mycobacterium tuberculosis or CMV infection. The susceptibility of recovered pregnant women to infections during pregnancy (which pertains to quantitative and qualitative loss of NK cell support), especially (but not exclusive) in countries where high-end laboratory techniques, such as flow cytometry or sequencing, are not routinely applied could be identified by skin prick test to measure inflammation as described by others (90). Furthermore, the disadvantages are false positivity, cost involved, and delayed results. Skin prick testing (SPT) has been shown to be safe, reliable and with high sensitivity and is the gold standard for the diagnosis of aeroallergens (89). Furthermore, SPT is minimally invasive and causes little discomfort. SPT have now been repurposed and could be deployed to measure inflammation in low-middle income countries for pregnant women (90), as they are relatively cheap and easy to interpret.

Taken together, our single-cell and FACS analyses indicate that multiple immune cell states and dynamic subpopulations underpinning the immune response during the window of infection and during recovery are deranged. Furthermore, analysis of a validation cohort highlighted the cellular complexity during infection and confirmed the preponderance of NK cell suppression in both the active and recovered phases. Moreover, our data constitute as a resource for the exploration of gene regulation during infection and benchmarking of genes/mechanistic studies *in vitro*. Our findings raise the possibility of developing a simple screening test to identify women at risk of infection and monitor the effectiveness of therapeutic interventions. Lastly, our study bolsters growing evidence of the mechanisms underlying post-COVID19 symptoms.

### Limitation of the study

Our results have added to the body of knowledge regarding SARS-CoV-2 infection during infection and provide key information for future research into the possible long-term effects post-infection. Although we carefully selected and characterized our patients for recruitment, the sample size was limited, as few pregnant women consented to donate blood samples for the transcriptomics study during the ongoing pandemic. Additionally, we were unable to recruit pregnant women with severe symptoms due to the lack of consent from these individuals or by their next of kin. We are mindful of the limitations of interpolating overexpressed or reduced RNA signatures, as pregnancy has a dynamic and evolving immune change at different stages of the trimester, as well as the impact of genetics and ancestry on our two cohorts. Furthermore, we acknowledge the limitations of using an *ex vivo* model that may not accurately recapitulate the *in vivo* situation. Longitudinal studies monitoring the PBMC composition intra-individually over time to capture the transition from a healthy state to a diseased state are required to provide useful information on immune dynamics and temporal shifts within each patient. Nonetheless, the impact of curtailed NK cell function post-infection on reproductive health and disease warrants further investigation.

## DeCOI consortium

DeCOI members are presented in https://decoi.eu/members-of-decoi/.

## Data Availability

The data generated by SC-RNA-sequencing from this study are available to download through the public repository via the following accession number or link: https://zenodo.org/records/14066080. Previous version of this manuscript is also available on bioRxiv: https://www.biorxiv.org/content/10.1101/2022.08.18.504053v1.full. The detailed analysis pipeline is available on github (https://github.com/ysinghbt/scPREG-R) used in this paper. Any additional information required to re-analyse the data reported in this paper is available from the lead contact upon reasonable request.

## Supplementary material

Supplementary Figure 1 Characterization of PBMCs from pregnant infected and recovered from SARS-CoV-2 infection. **(a)** From left to right: Gating strategy for the 14-colour flow cytometry panel. Based on FSC and SSC we first removed the cell debris. FSC-A and FSC-W was used to remove the doublets and focussed on single cells. In the next step, we removed the dead cells using Violet Live/Dead staining. Finally, we used FSC-A vs SSC-A to gate lymphocytes and monocytes based on size and granularity. Lymphocytes were discriminated in CD19 and CD3 based on cell surface markers and CD3 cells were discriminated into CD4 and CD8 cells using CD4 and CD8a antibodies. **(b)** Original FACS plots. SC-A vs SSC-A show the monocytes and lymphocyte gated population (FACS plots) for Preg-HC, Preg- INF and Preg-R groups. **(c)** The percentage of lymphocytes shown by violin plots for Preg-HC, Preg-INF and Preg-R samples. Kruskal-Wallis nonparametric test and multiple comparisons based on *post-hoc* Dunn’s test was employed to compare three groups - Preg-HC, Preg-SARS-CoV-2 and Preg-R. P value ≤0.05 is considered significant. **(d)** The percentage of monocytes shown by violin plots (lower side) for Preg-HC, Preg-INF and Preg-R samples. Kruskal-Wallis nonparametric test and multiple comparisons based on *post-hoc* Dunn’s test was employed to compare three groups - Preg-HC, Preg-SARS-CoV-2 and Preg-R. P value ≤0.05 is considered significant. **(e)** Original FACS plots for CD4 versus CD8a staining gated on CD3^+^ T cells. **(f)** The percentage of CD4^+^ T cells in Preg-HC, Preg-INF and Preg-R samples. **(g)** The percentage of CD8^+^ T cells in Preg-HC, Preg-INF and Preg-R samples.

Supplementary Figure 2 Decreased NK cells in infected and recovered pregnant women. **(a)** The percentage of CD3^-^CD19^-^CD56^+^HLA-DR^-^ NK cells shown in FACS plots (upper panel) and violin plots (lower panel) in Preg-HC, Preg-INF and Preg-R samples. **(b)** The percentage of CD3^-^CD19^-^HLA-DR^-^CD56^+^ NK cells and CD3^-^CD19^-^HLA-DR^+^CD56^+^ NK cells are shown in violin plots. No statistical significance was observed, however, low abundance of CD3^-^CD19^-^HLA-DR^-^CD56^+^ NK cells were noticed in Preg-HC and Preg-R groups. **(c)** UMAP analysis of CD3^-^CD19^-^ cells for different subsets of NK cells based on CD56 and CD16 expression for the terminal (NKIII, blue), mature (NK II, green), early NK (NKI, pink) and non-NK cells (red), while grey coloured cells show the all the samples containing NK cells. **(d)** Overlay of UMAP to show the Preg-HC (blue), Preg-R (navy) and Preg-D (red) for NK cells. All the combined samples were shown in background (grey). **(e)** FACS plots of CD3^-^CD19^-^CD56^+^HLA-DR^-^ cells for different subsets of NK cells based on CD56 and CD16 expression for the terminal (NKIII), classical/mature (NK II), early NK (NKI) and non-NK cells. **(f)** The percentage of different subsets of NK cells based on CD56 and CD16 makers for CD3^-^CD19^-^CD56^+^HLA-DR^-^ cells. The violin plots of classical (NKII) and non-NK cells.

Supplementary Figure 3 Examination of inflammatory cytokines and covid-19 antibody levels in the serum of recovered women up to 89 days post infection. **(a)** Nucleocapsid IgG levels in Preg-R individuals at different collection time points; CT1, CT2 and CT3). **(b)** Significantly increased levels of cytokines (pg/mL) IFN-γ, IL1β, TNFα, IL6, IL12p70, IL23 and IL33 in Preg-R patients. No significant change was noticed in IFN-α2 levels. Each dot represents an individual. Kruskal-Wallis test, adjusted for Dunn's multiple comparisons test. P value ≤ 0.05 considered significant (*p ≤ 0.05, **p ≤ 0.01, *** p ≤ 0.001, ****p ≤ 0.0001).

Supplementary Figure 4 Quality check and UMAP analysis for single cell RNA-seq data from healthy control and recovered pregnant women. **(a)** Total 46,594 cells were recovered after sequencing from 8 samples (Preg-HC and Preg-R). First RNA-sequenced cells were gated for genes expressed vs library size; we found 30,394 high quality cells. Next high QC cells were analysed for cells expressing based on total read cut off min cells 5 were chosen for further analysis. Cells expressing highly dispersed gene were gated and used for PCA and UMAP analyses. Violin plots showing the nFeature, nCounts, percentage mitochondria and percentage of ribosomes before filtering (left panel). **(b)** Violin plots show the nFeature, nCounts, percentage mitochondria and percentage of ribosomes post-filtering (right panel). **(c)** Pre-integration of all the samples showed on UMAP plots. **(d)** Post-integration of all the samples showed on UMAP plots.

Supplementary Figure 5 Identification of cell clusters. **(a)** Dot plot represents identification of different cell subsets based on canonical markers. **(b)** Top 10 genes were used for identification different cell clusters and presented as a heatmap.

Supplementary Figure 6 Deregulated B cells in Preg-R **(a)** UMAP plots for B cell clusters including both naïve (C10) and memory cells (C16). **(b)** Differential gene expression analysis of naïve and memory B cells to identify the genes in Preg-R. Most significantly changed genes are shown in the volcano plot in red (up and down). **(c)** GSEA enrichment pathways analyses of naïve and memory B cells. **(d)** Regulation of interleukin-8 production pathway was downregulated in naïve B cells in Preg-R group patients. **(e)** Downreguled type 1 interferon-mediated signalling, IL-4 & IL-8 production pathways whilst natural killer cell activation pathways were activated in B memory cell of Preg-R women.

Supplementary Figure 7 Deregulated CD4^+^ T cell subsets in Preg-R **(a)** UMAP plots for CD4^+^ naïve (C0) and CD4^+^ CTLs (C5) T cell clusters. **(b)** Differential gene expression analysis in Preg-R of (C0) and CD4^+^ CTLs (C5). Most significantly changed genes are shown oin the volcano plot in red (up and down). **(c)** Dot plot presents top 10 upregulated and downregulated genes in CD4^+^ T cell subsets in Preg-HC and Preg-R groups. Selected up and downregulated genes in different subsets of T cells; CD4^+^ naïve (C0), CD4^+^ CM (C4), CD4^+^ CTLs (C5), CD4^+^ EM (C8), CD4^+^ TEMRA (C13), CD4^+^ Tregs (C17) (upper dot plot). Violin plots show significant down regulation of IRF9, IRF1 and RORA expression in Preg-HC vs Preg-HC (lower violin plots). P value significance was calculated based on Seurat. **(d)** GSEA enrichment pathways analyses of naïve and CD4^+^ CTLs. **(e)** Natural killer cell activation pathways were activated in CD4^+^ Naïve T cells (Left). Further, CD4^+^ CTLs had activated activities of histone H3 methyltransferase activity, post-transcriptional gene silencing, T cell differentiation, and Th-17 immune response.

Supplementary Figure 8 Deregulated MAIT cells response in Preg-R **(a)** UMAP plots for MAIT cells (left UMAP plot). Differential gene expression analysis in Preg-R of MAIT cells (right; volcano plot). Most significantly genes are shown on the volcano plot in red (up and down). **(b)** GSEA pathways analysis of MAIT cells. Selected activated and suppressed pathways are shown in bubble plot. **(c)**. Enrichment GSEA plots show activated immune response-regulating signalling and suppressed reactive oxygen species metabolic process pathways in MAIT cells.

Supplementary Figure 9 Expression of CD215 on monocytes. FACS histogram plot present the expression of CD215 on CD14^+^ Monocytes. Preg-HC and Preg-R samples overlayed on each other for identification of CD215 expression for comparative purpose and expression was normalized to mode (left FACS plot). Box and Whisker plot represents the median expression of CD215 marker.
