# Supplementary figures and images for: Single-cell RNA-sequencing highlights a curtailed NK cell function in convalescent COVID-19 pregnant women
Source: Front Immunol. 2025 Jun 30;16:1560391. doi: 10.3389/fimmu.2025.1560391 (PMC12257036; doi:10.3389/fimmu.2025.1560391)

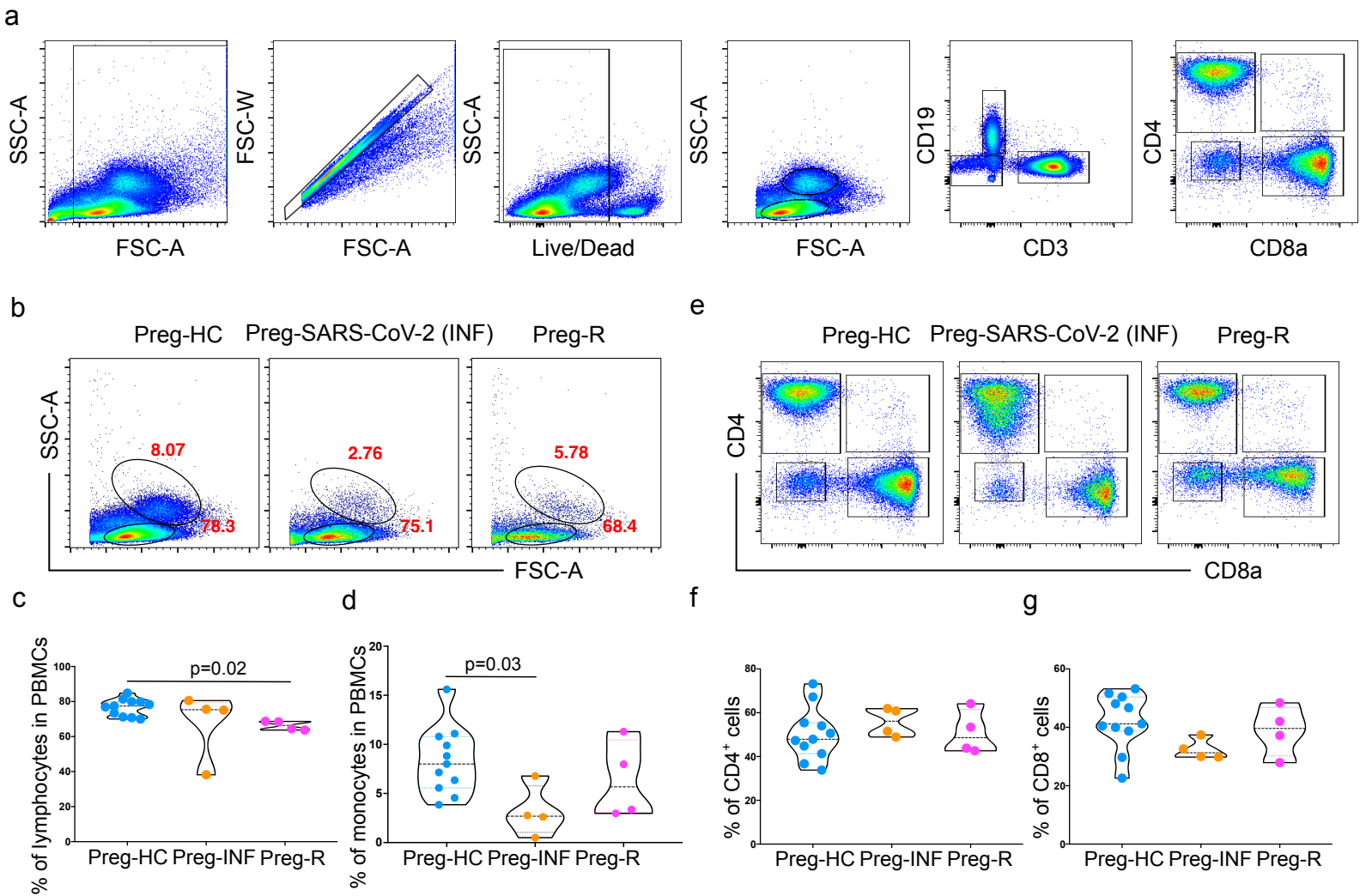

Suppl. Fig. 1

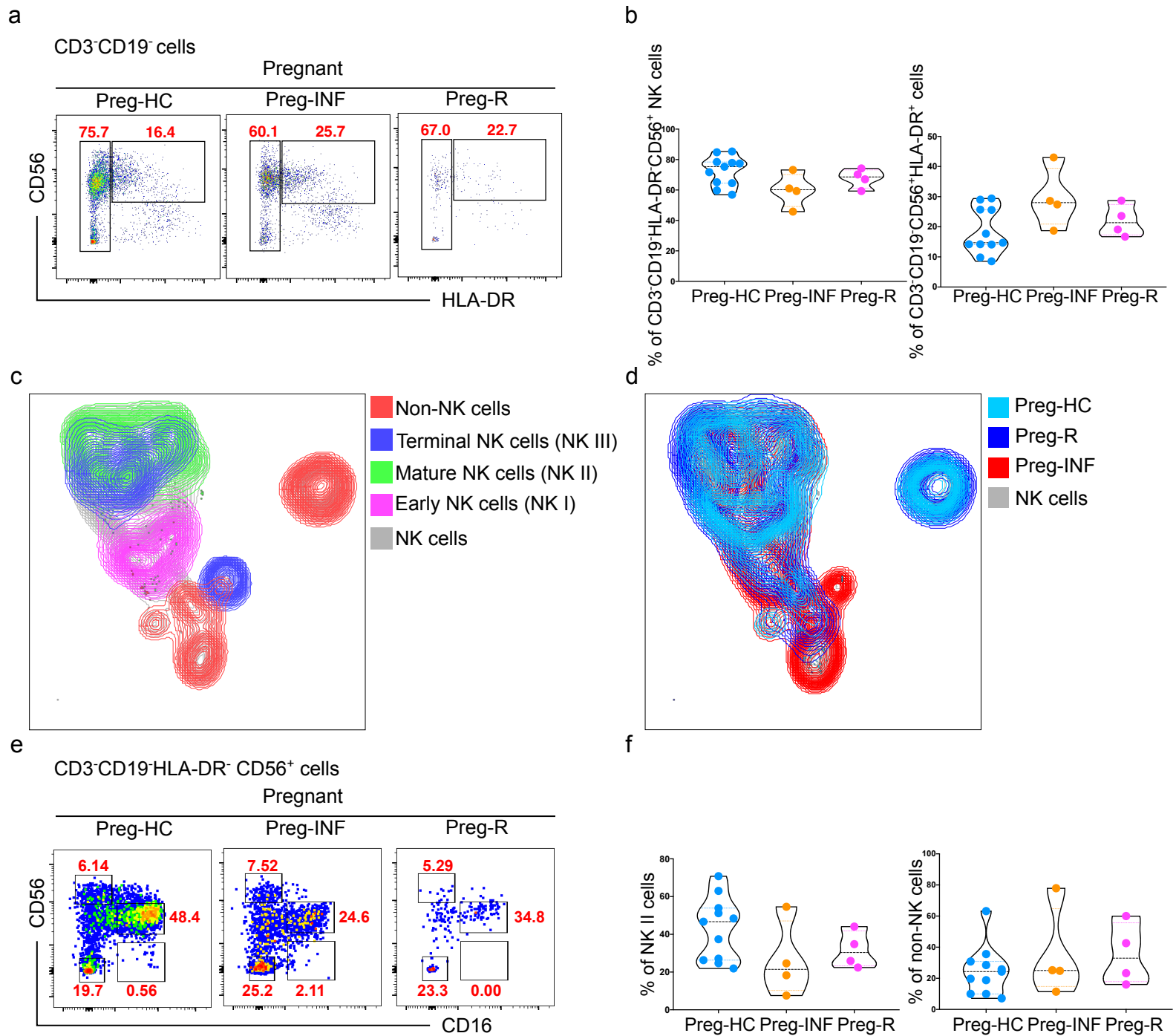

Suppl. Fig. 2

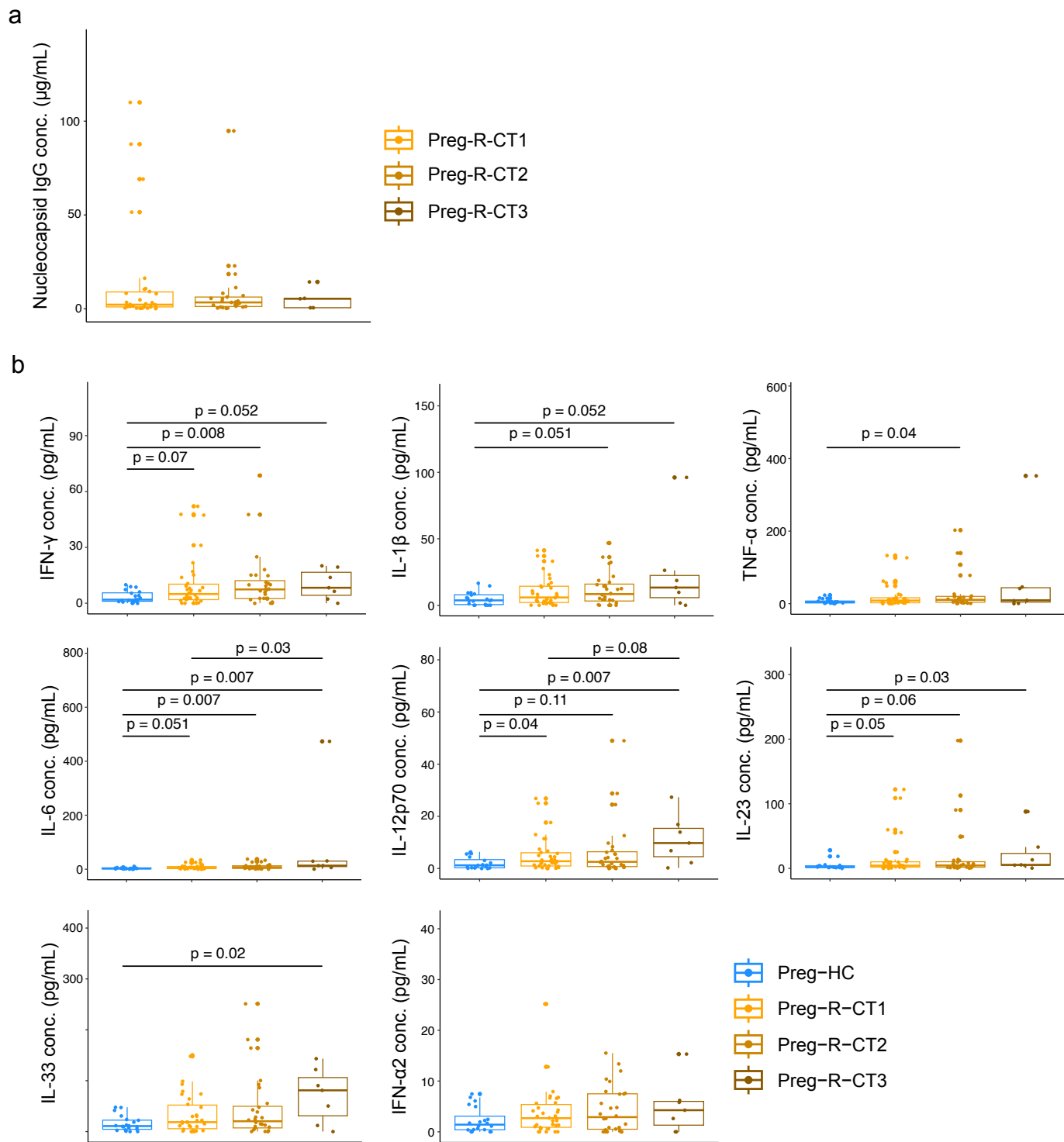

Suppl. Fig. 3

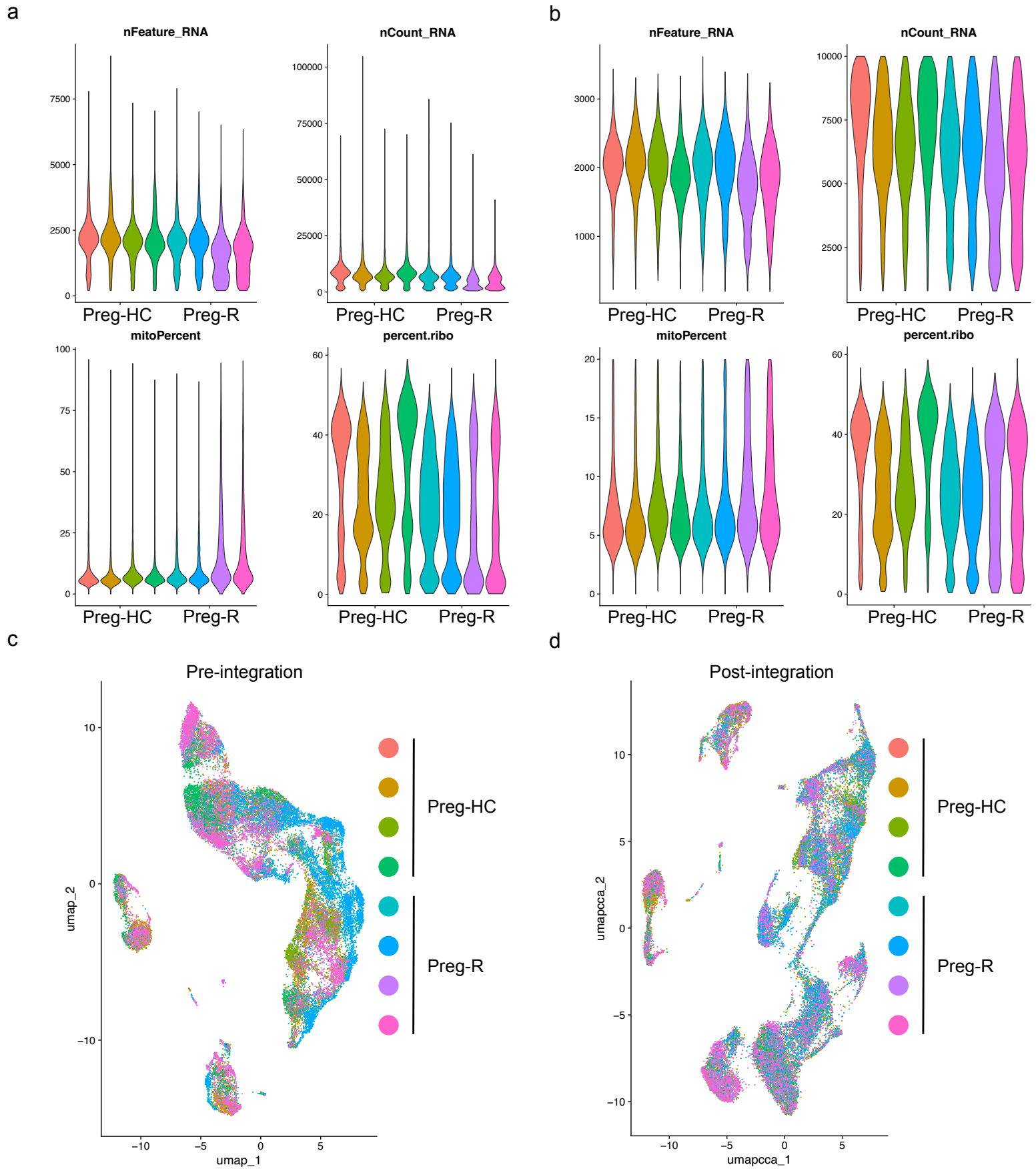

Suppl. Fig. 4

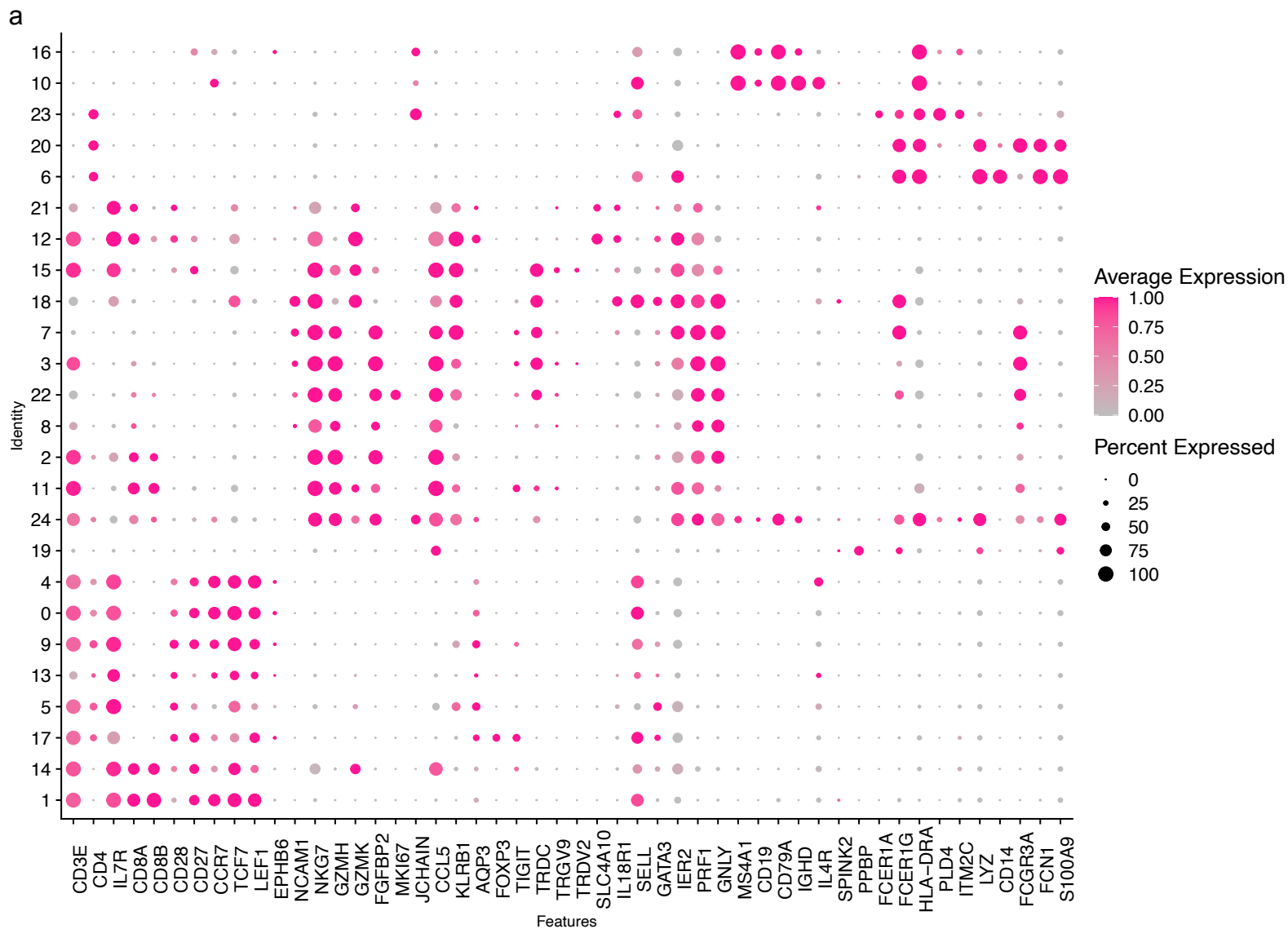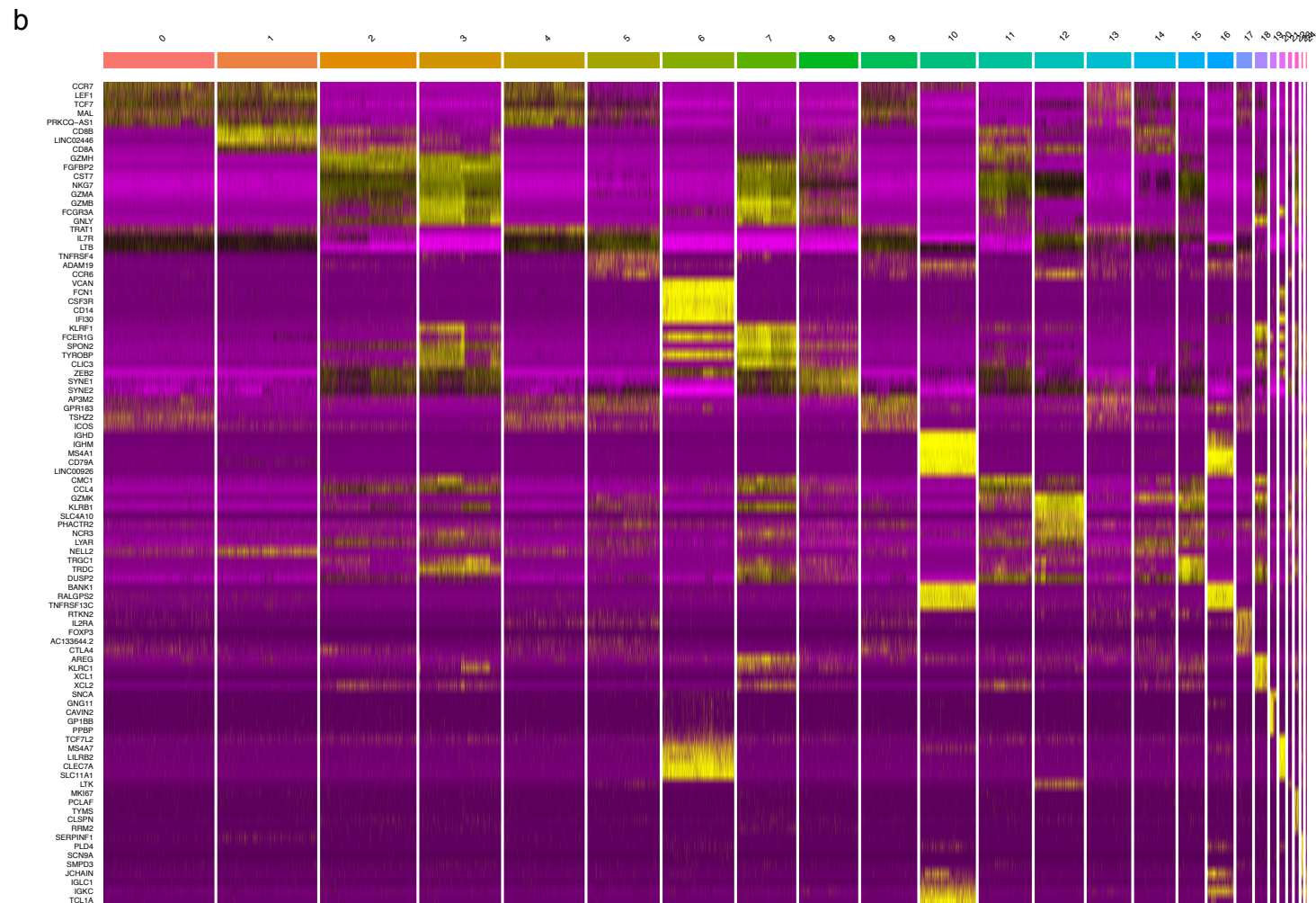

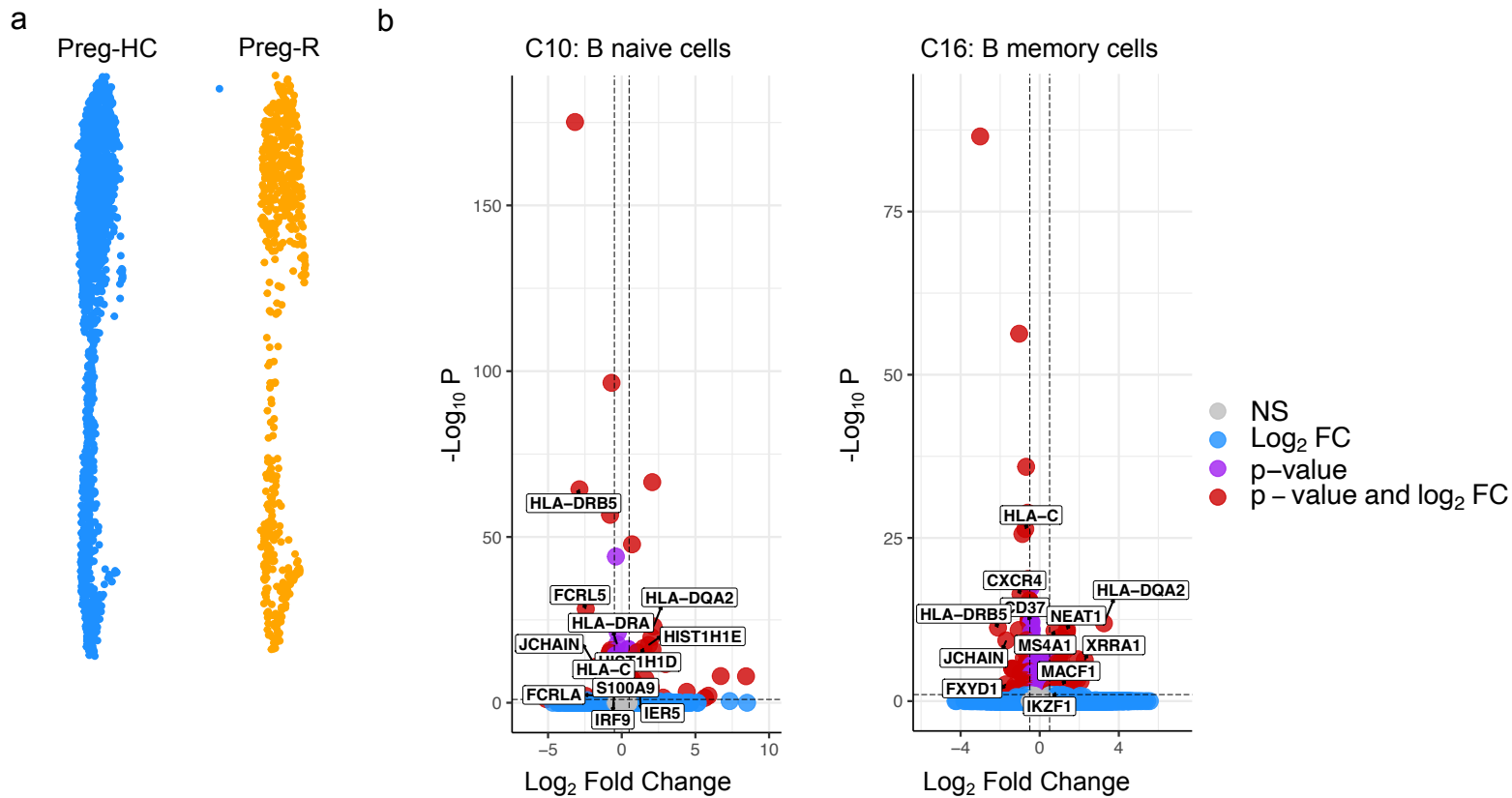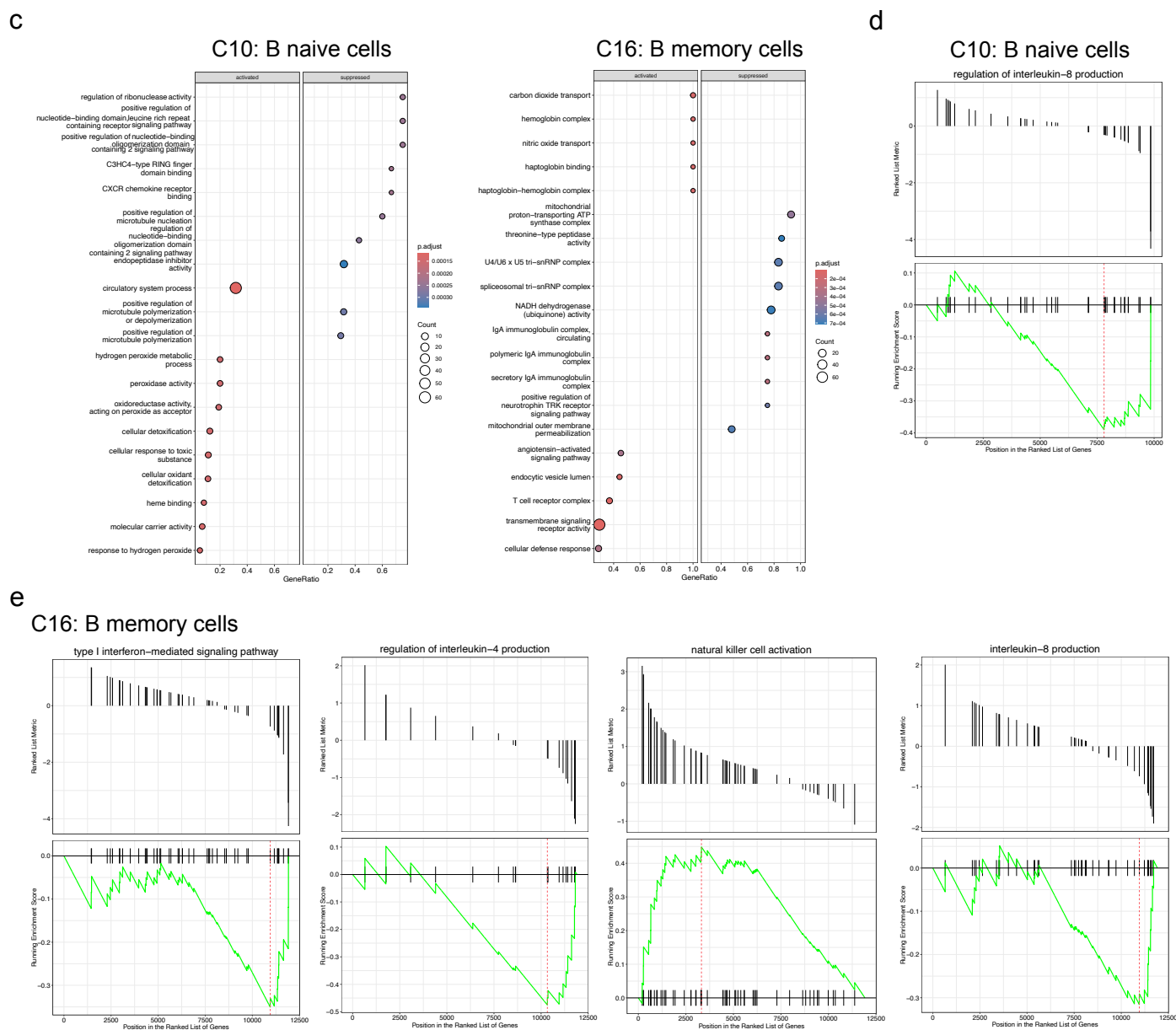

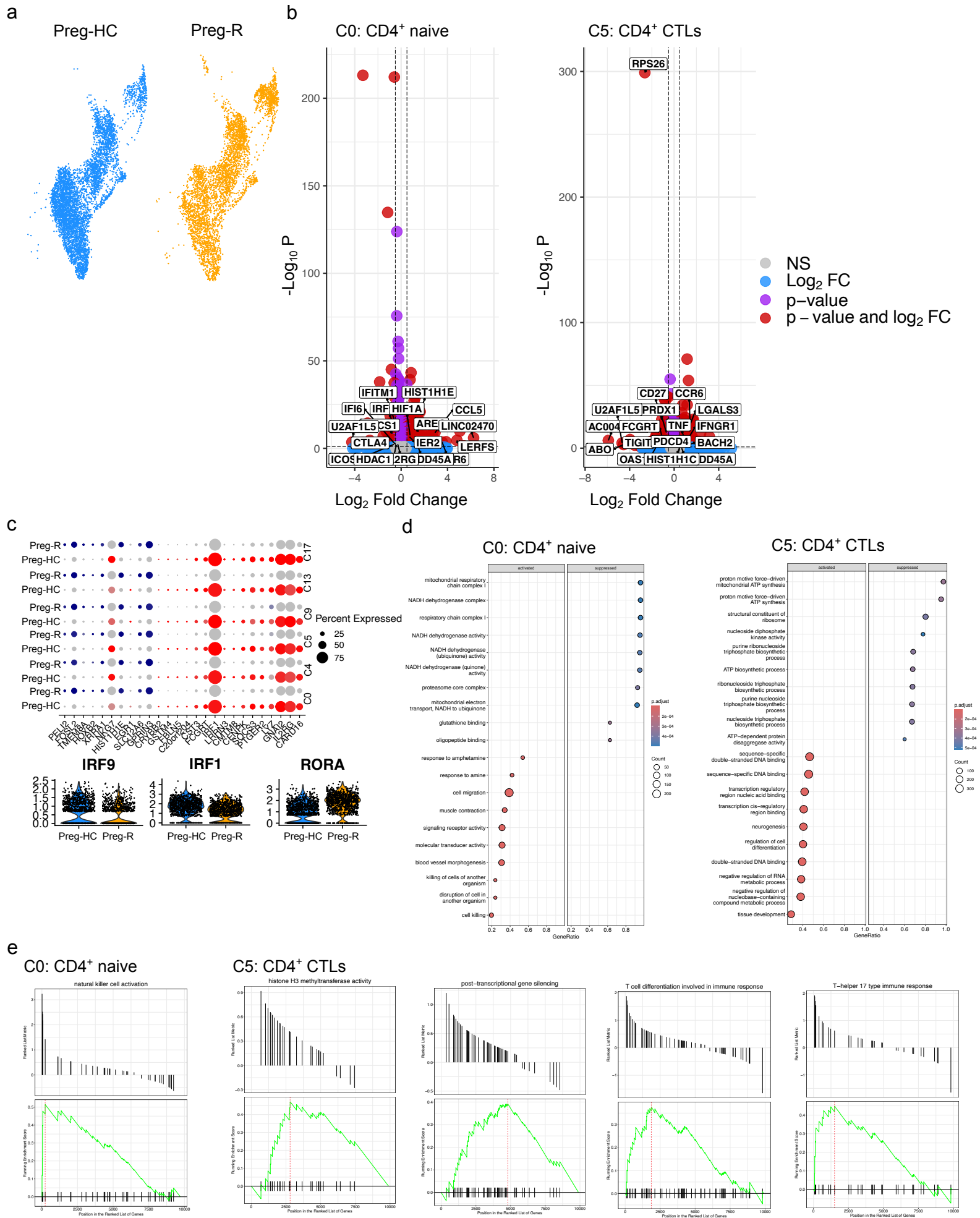

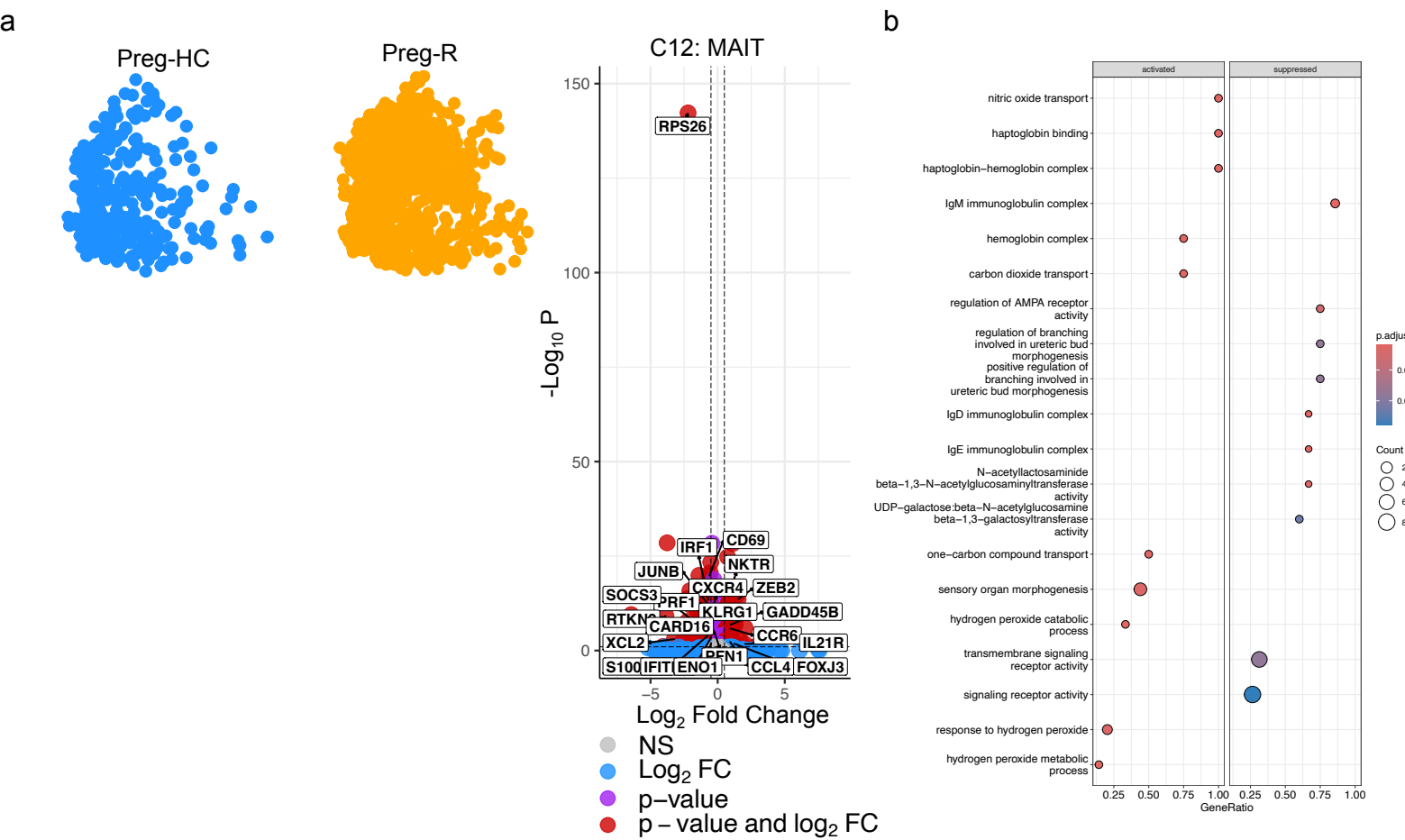

Suppl. Fig. 8

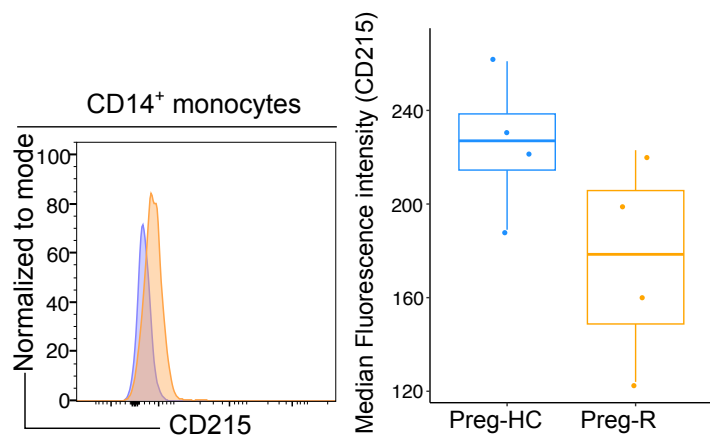

Suppl. Fig. 9

Supplement: Supplementary Figure 1 — Characterization of PBMCs from pregnant infected and recovered from SARS-CoV-2 infection. (a) From left to right: Gating strategy for the 14-colour flow cytometry panel. Based on FSC and SSC we first removed the cell debris. FSC-A and FSC-W was used to remove the doublets and focussed on single cells. In the next step, we removed the dead cells using Violet Live/Dead staining. Finally, we used FSC-A vs SSC-A to gate lymphocytes and monocytes based on size and granularity. Lymphocytes were discriminated in CD19 and CD3 based on cell surface markers and CD3 cells were discriminated into CD4 and CD8 cells using CD4 and CD8a antibodies. (b) Original FACS plots. SC-A vs SSC-A show the monocytes and lymphocyte gated population (FACS plots) for Preg-HC, Preg- INF and Preg-R groups. (c) The percentage of lymphocytes shown by violin plots for Preg-HC, Preg-INF and Preg-R samples. Kruskal-Wallis nonparametric test and multiple comparisons based on post-hoc Dunn’s test was employed to compare three groups - Preg-HC, Preg-SARS-CoV-2 and Preg-R. P value ≤0.05 is considered significant. (d) The percentage of monocytes shown by violin plots (lower side) for Preg-HC, Preg-INF and Preg-R samples. Kruskal-Wallis nonparametric test and multiple comparisons based on post-hoc Dunn’s test was employed to compare three groups - Preg-HC, Preg-SARS-CoV-2 and Preg-R. P value ≤0.05 is considered significant. (e) Original FACS plots for CD4 versus CD8a staining gated on CD3+ T cells. (f) The percentage of CD4+ T cells in Preg-HC, Preg-INF and Preg-R samples. (g) The percentage of CD8+ T cells in Preg-HC, Preg-INF and Preg-R samples. [file DataSheet1.pdf]
